# BinPacker: Packing-Based *De Novo* Transcriptome Assembly from RNA-seq Data

**DOI:** 10.1371/journal.pcbi.1004772

**Published:** 2016-02-19

**Authors:** Juntao Liu, Guojun Li, Zheng Chang, Ting Yu, Bingqiang Liu, Rick McMullen, Pengyin Chen, Xiuzhen Huang

**Affiliations:** 1 School of Mathematics, Shandong University, Jinan, China; 2 High Performance Computing Center, University of Arkansas, Fayetteville, Arkansas, United States of America; 3 Crop, Soil, and Environmental Sciences, University of Arkansas, Fayetteville, Arkansas, United States of America; 4 Department of Computer Science, Arkansas State University, Jonesboro, Arkansas, United States of America; Max-Planck-Institut für Informatik, GERMANY

## Abstract

High-throughput RNA-seq technology has provided an unprecedented opportunity to reveal the very complex structures of transcriptomes. However, it is an important and highly challenging task to assemble vast amounts of short RNA-seq reads into transcriptomes with alternative splicing isoforms. In this study, we present a novel *de novo* assembler, BinPacker, by modeling the transcriptome assembly problem as tracking a set of trajectories of items with their sizes representing coverage of their corresponding isoforms by solving a series of bin-packing problems. This approach, which subtly integrates coverage information into the procedure, has two exclusive features: 1) only splicing junctions are involved in the assembling procedure; 2) massive pell-mell reads are assembled seemingly by moving a comb along junction edges on a splicing graph. Being tested on both real and simulated RNA-seq datasets, it outperforms almost all the existing *de novo* assemblers on all the tested datasets, and even outperforms those *ab initio* assemblers on the real dog dataset. In addition, it runs substantially faster and requires less memory space than most of the assemblers. BinPacker is published under GNU GENERAL PUBLIC LICENSE and the source is available from: http://sourceforge.net/projects/transcriptomeassembly/files/BinPacker_1.0.tar.gz/download. Quick installation version is available from: http://sourceforge.net/projects/transcriptomeassembly/files/BinPacker_binary.tar.gz/download.

## Author Summary

The availability of RNA-seq technology drives the development of algorithms for transcriptome assembly from very short RNA sequences. However, the problem of how to (*de novo*) assemble transcriptome using RNA-seq datasets has not been modeled well; e.g. sequence coverage information has even not been accurately and effectively integrated into the appropriate assembling procedure, leading to a bottleneck that all the existing (*de novo*) strategies have encountered. We present a novel approach to remodel the problem as tracking a set of trajectories of items with their sizes representing the coverage of their corresponding isoforms by solving a series of bin-packing problems. This approach, which subtly integrates the coverage information into the procedure, has two exclusive features: 1) only splicing junctions are involved in the assembling procedure; 2) massive pell-mell reads are assembled seemingly by moving a comb along junction edges on a splicing graph. Being tested on both real and simulated RNA-seq datasets, it outperforms almost all existing *de novo* assemblers on all the tested datasets, even outperforms those *ab initio* assemblers on the dog dataset, in terms of commonly used comparison standards.

This is a *PLOS Computational Biology* Methods paper.

## Introduction

The advent of RNA-seq techniques are changing how transcription, splicing variations and associated mechanisms can be studied since they provide unprecedented accuracy about the mRNA expression level [1]. They allow accurate elucidation of all splicing variants, including the rare and lowly expressed splicing isoforms. This clearly opens many new doors for studying the mechanisms of various human diseases that are related to abnormal splicing [1], including cancers. With the RNA-seq techniques, there come new challenges associated with the interpretation of the generated datasets. Although sequencing reads from PacBio RS II sequencer are long enough to cover multiple exons, they have not been commonly used to improve the state of the art transcripts reconstruction because they are suffering from higher error rates [2]. Therefore the RNA-seq techniques for short sequencing reads [3] remain necessary. One major challenge is how to accurately assemble the short sequencing reads into full-length transcripts possibly involving multiple splicing variants, the so-called RNA-seq based transcriptome assembly problem.

According to the literatures [4–6], there are various alternative splicing events capable of producing multiple isoforms in eukaryotic genes. Event types include skipped exons, retained introns and mutually exclusive exons. Even more complicated, some exons may be partially involved in transcripts during the alternative splicing process. At first glance, the transcriptome assembly is similar to genome assembly, but they are actually fundamentally different. In contrast, the following facts make the transcriptome assembly more challenging: (i) some transcripts have a very low expression level, while others may be expressed in a dramatically high level [7]; (ii) each locus usually produces multiple transcripts due to various alternative splicing events [8]; (iii) some transcripts with low expression level may be submerged due to the sequencing errors [8,9]. Therefore, a successful transcriptome assembler should overcome all these difficulties, and be capable of recovering all full-length transcripts of variable lengths, expression levels and noises.

Computational strategies for transcriptome assembly can be generally divided into two categories, *ab initio* and *de novo* [1,8]. If a reference genome is available, *ab initio* approaches, such as Cufflinks [10] and Scripture [11], usually start by mapping RNA-Seq reads to the reference genome, and then sequences with overlapping alignment are merged into a connectivity graph on which the well studied min-cost minimum path cover model is subtly employed to extract a minimum set of paths which explain the RNA-seq dataset. A very recently published *ab initio* assembler, StringTie [12], also first maps RNA-Seq reads to the reference genome, then constructs alternative splicing graphs and then assembles transcripts by using a maximum-flow network model. *De novo* approaches, such as ABySS [13], SOAPdenovo-Trans [14], Oases [15] and IDBA-Tran [16], directly use the reads to assemble transcripts, without mapping them to a reference genome, which is important when the reference genome is unavailable, incomplete, highly fragmented or substantially altered as in cancer tissues. These *de novo* approaches which were developed based on the techniques used in genome assembly are not solving all the transcriptome assembly problems in general [7]. Trinity [8] which was designed specifically for *de novo* transcriptome assembly has substantially improved the state of the art *de novo* transcriptome assemblers. It starts by extending short reads through overlaps into contigs, connecting contigs into a graph, and then extracts paths from this graph to construct splicing variants based on a brute-force enumeration strategy. Trinity does improve previous *de novo* assemblers which have their roots in genome assembly techniques, but it does not introduce an appropriate model to optimize its solution, and even not incorporate sequencing coverage depth information into the assembly procedure either, although the authors in Trinity have noticed that similarity of the coverage depth across different coding regions in a transcript could be useful. To this end, we have recently presented a new *de novo* transcriptome assembler, Bridger [17], which “bridges” between Cufflinks and Trinity so that the techniques used in Cufflinks can be employed to overcome the limitations of Trinity. Bridger does incorporate the coverage information into the assembly procedure via an appropriate model, but it could not guarantee a genuine solution due to (1) in-weight and out-weight are defined somewhat arbitrarily in Bridger; (2) a node with both in-edges and out-edges has no chance to be an end of any transcripts. Therefore, there still remains room for improvement.

In this paper we develop a novel *de novo* algorithm, BinPacker, to assemble full-length transcripts by remodeling the problem as tracking a set of trajectories of items over a splicing graph, which is constructed by employing the techniques used in Bridger [17] with several updates described in Methods. The set of trajectories of items over the splicing graph can be achieved by solving a series of variants of the bin-packing problem, which are different from the traditional bin-packing problem, which is defined to pack a given number of items of different sizes into as few bins of a given size as possible, and each bin can only hold items with the sum of their sizes no more than the size of the bin. We have tested and compared BinPacker with seven competitive *de novo* assemblers, Trinity [8], ABySS [13], Trans-ABySS [18], SOAPdenovo-Trans [14], Oases [15], IDBA-Tran [16] and Bridger [17] on real and simulated datasets. The simulation dataset is generated as described in Results section. For the real datasets, three datasets are used, including two standard RNA-seq datasets, one dog and one human, and one strand-specific mouse RNA-seq dataset. The comparison results show that BinPacker outperforms almost all the compared assemblers on all datasets, including real and simulated, in terms of commonly used standards for evaluation of transcriptome assemblers. Even more surprisingly, it outperforms StringTie, a most recently published *ab initio* assembler [12], on dog dataset.

## Results

We ran BinPacker, and seven other *de novo* assemblers: ABySS (version 1.3.4), Trans-ABySS (version 1.4.4), Trinity (version 2012-10-05), Velvet (version 1.2.01) + Oases (version 0.2.02), SOAPdenovo-Trans (version 1.01), IDBA-Tran (version 1.1.1), and Bridger, and also the most recently published *ab initio* assembler StringTie on real and simulated datasets below, and tested their performance with the optimized parameters on the same server with 512GB of RAM (see S1 Text for details).

The criteria that have previously been used to test the assemblers are employed in our testing. All assembled transcripts are matched against all known transcripts in the annotation (referred to as ‘‘reference transcripts”) using BLAT [19], with 95% identity as the cutoff. If an assembled transcript full-length covers a reference transcript with at least 95% sequence identity and at most 0.5% indels, this reference transcript is counted as full-length recovered, and noted as a true positive. The indel cutoff is used to avoid the over-estimated consistencies between the predicted and the references. In this paper, sensitivity is defined as the number of full-length recovered reference transcripts, and accuracy is defined as the true positive rate. We further consider two types of accuracy. One is related to a reference true positive rate which is the rate between the number of full-length recovered reference transcripts and the number of assembled transcripts, and the other is related to an assembled true positive rate which is the fraction of assembled transcripts that are in the reference transcripts. The reliability of an assembler is defined by the distribution of its recovered reference transcripts against recovered sequence length rates ranging from 80% to 100%. An assembler is considered of higher reliability if it recovers more reference transcripts with recovered sequence length rates ranging from 90% to 100%.

### 1. Tests on real datasets

We ran and tested all the 9 assemblers on three real RNA-seq datasets which include two standard (non-strand specific) Illumina datasets from dog and human, and one strand-specific dataset from mouse.

#### 1.1. Collection of real datasets

The dog dataset was collected from NCBI SRA database (Accession Code: SRR882093), the human dataset was collected from the DDBJ SRA database (Accession Codes: SRX011545 and SRX011546) and the mouse dataset was collected from C567BL/6 mouse primary immune dendritic cells (Accession Code: SRX062280 in the DDBJ SRA database). The reference transcripts of dog were downloaded from UCSC [20]. The human and mouse reference transcripts were downloaded from Ensemble Genome Browser [21].

#### 1.2. Comparing BinPacker to the other assemblers on real datasets

We compare BinPacker to the other assemblers on the real datasets mentioned above in terms of sensitivities, accuracies and their distributions against recovered sequence length rates ranging from 80% to 100%.

**Fig 1:**
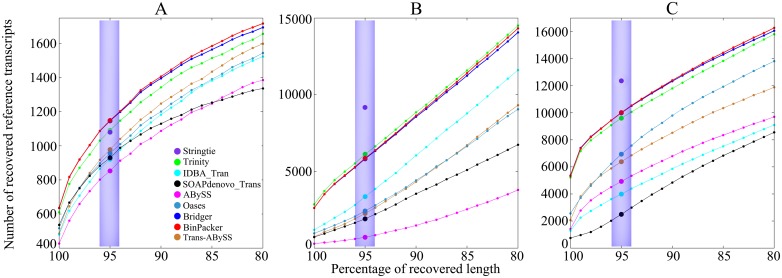
Comparison of recovered reference sensitivity and its distribution against recovered sequence length rates (sequence identity) ranging from 80% to 100% on (A) dog, (B) human and (C) mouse datasets. Solid colored circles in shaded areas represent the number of full-length recovered reference transcripts for different assemblers.

*1.2.1 Comparison of sensitivities and their distributions against recovered sequence length rates on dog and mouse datasets*. We run all the *de novo* assemblers on dog and mouse datasets. The results show that BinPacker reaches the highest sensitivity, recovering 1149 and 10012 full-length transcripts among 33665 and 39060 candidates respectively on dog and mouse datasets, while Trinity recovers 1091 and 9599 among 49311 and 78333, and Bridger recovers 1147 and 9991 among 37234 and 50051. Bridger performs a little worse than BinPacker, but better than Trinity and all the other *de novo* assemblers (Fig 1A and 1C, shaded area). Trinity performs worse than BinPacker because it uses an exhaustive enumeration algorithm to search for paths in *de Bruijn* graphs without using sequencing depth information in the searching process, which results in the increase of false positives and the decrease of true positives. Bridger performing worse than BinPacker is due to the facts: 1) the weights in compatibility graph are defined a bit arbitrarily, and 2) a node with both in-edges and out-edges in the splicing graph will never be an end of a transcript. Apart from the three best *de novo* assemblers mentioned above, Trans-ABySS performs best on dog dataset, while Oases does best on mouse dataset. We further compared BinPacker with StringTie, a most recently published *ab initio* assembler. As expected, StringTie performs best on mouse dataset. Surprisingly, while it is defeated by BinPacker on dog dataset, StringTie recovers 1072 full-length transcripts, compared to 1149 recovered by BinPacker.

To test the reliability of these *de novo* assemblers, we compare their sensitivity distributions against recovered sequence length rates ranging from 80% to 100%. As shown in Fig 1A and 1C, BinPacker keeps the highest sensitivity in the whole interval [80%, 100%] on both dog and mouse datasets. Bridger's sensitivity is a little lower than BinPacker's, while Trinity is lower than both BinPacker and Bridger, but higher than the others in the whole interval [80%, 100%].

**Fig 2:**
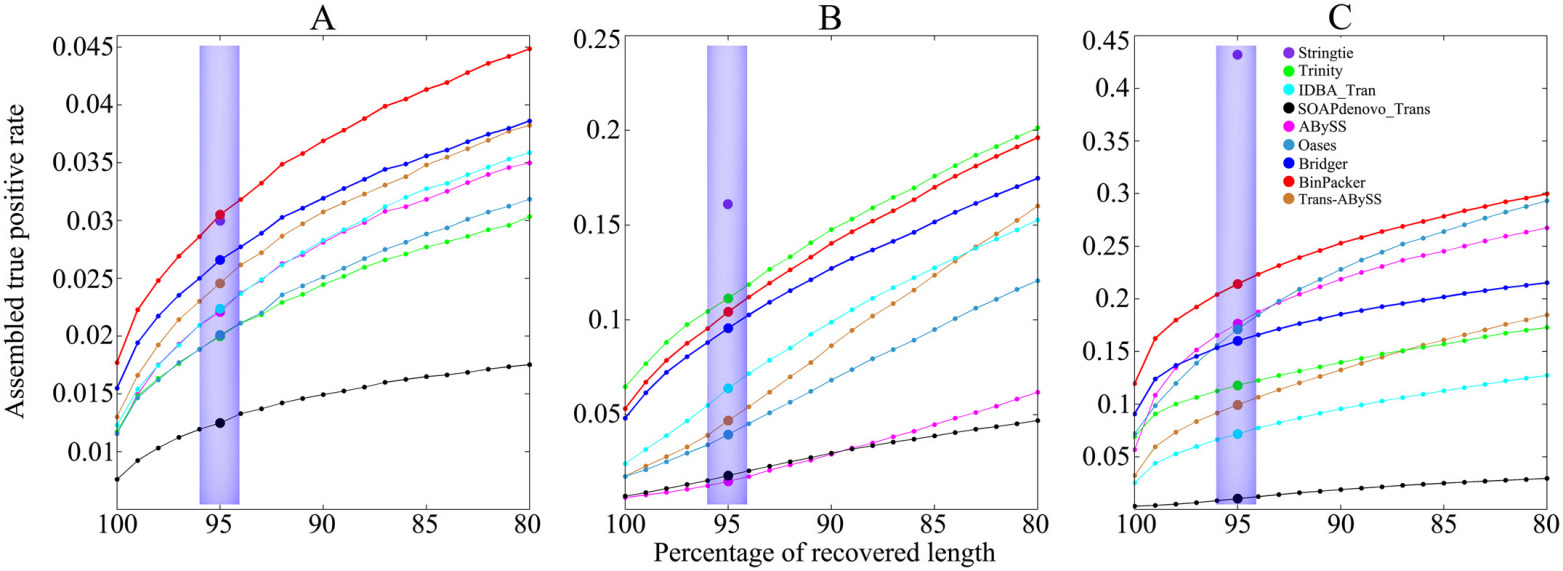
Comparison of assembled true positive rate and its distribution against recovered sequence length rates (sequence identity) ranging from 80% to 100% on (A) dog, (B) human and (C) mouse datasets. Solid colored circles in shaded areas represent the assembled true positive rate for different assemblers.

**Fig 3:**
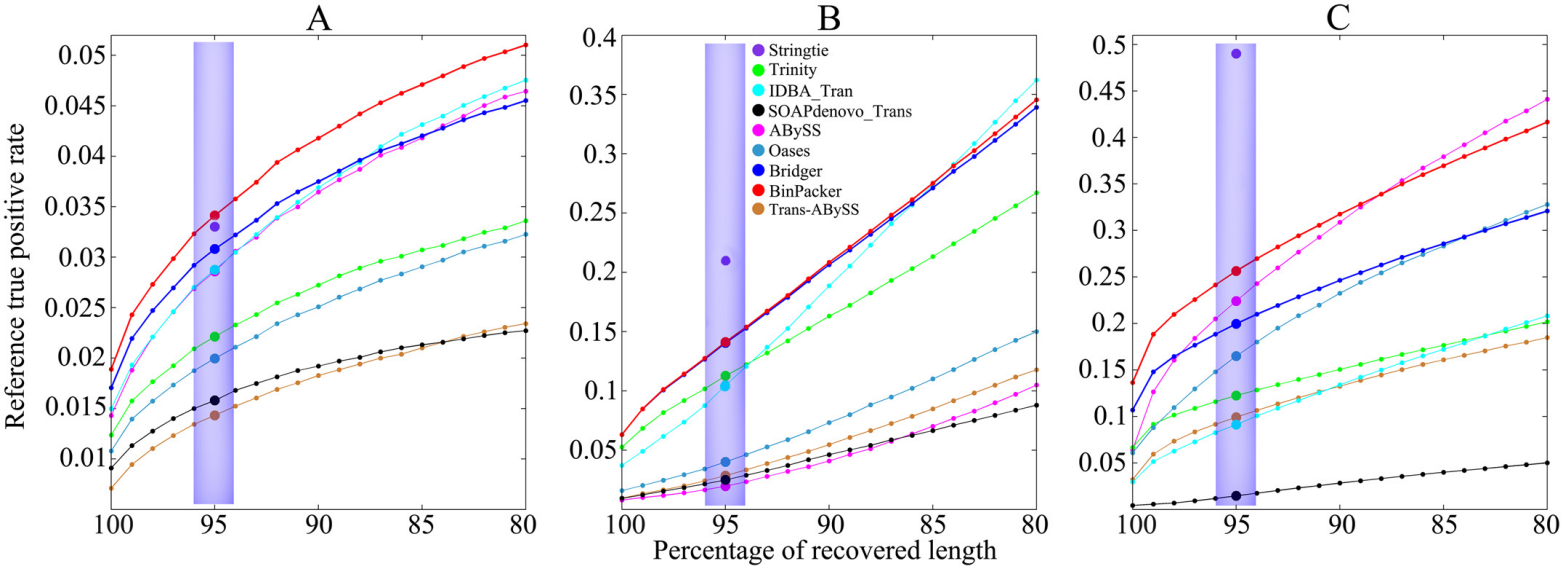
Comparison of reference true positive rate and its distribution against recovered sequence length rates (sequence identity) ranging from 80% to 100% on (A) dog, (B) human and (C) mouse datasets. Solid colored circles in shaded areas represent the reference true positive rate for different assemblers.

*1.2.2 Comparison of accuracies and distributions against recovered sequence length rates on dog and mouse datasets*. Our comparison results show that BinPacker outperforms all the other *de novo* assemblers we are comparing with in terms of both types of accuracy on dog and mouse datasets (Figs 2A and 2C and 3A and 3C, shaded area). Of all the other assemblers, Bridger performs best on dog dataset in terms of both types of accuracy, while ABySS performs best on mouse dataset in terms of both types of accuracy. Trinity suffers from very low accuracy on both dog and mouse datasets because of its large false positives. StringTie as expected performs best on mouse dataset, but unexpectedly worse than BinPacker on the dog dataset in terms of both types of accuracy.

The comparison results of accuracy distributions against recovered sequence length rates ranging from 80% to 100% (Figs 2A and 2C and 3A and 3C) show that BinPacker keeps the highest accuracy level in the interval [90%, 100%] on both dog and mouse datasets. The following are some details of the performances of the other assemblers excluding BinPacker. Bridger keeps the highest accuracy level among the others on dog dataset in terms of both types of accuracy in the interval [90%, 100%]. On the mouse dataset, ABySS keeps the highest among the others excluding Bridger in terms of reference true positive rate in the interval [90%, 100%], and Oases keeps the highest among the others excluding Bridger in terms of assembled true positive rate in the interval [80%, 100%] excluding [94%, 99%]. Trinity again loses in accuracy of both types in the interval [80%, 100%] on both dog and mouse datasets.

Therefore we conclude that BinPacker has the highest reliability among all the *de novo* assemblers we are comparing with in terms of their distributions of sensitivity and accuracy against recovered sequence length rates on real dog and mouse datasets.

*1.2.3 BinPacker maintains a stable performance on human dataset*. The human dataset is also adopted to test the performance of BinPacker and the other assemblers. The results show that BinPacker outperforms all the other *de novo* assemblers except Trinity on some cases. The following are some details. For the sensitivity, BinPacker, Bridger and Trinity recovered 5859, 5822 and 6122 full-length reference transcripts from 41691, 41470 and 54315 candidates, respectively. StringTie recovered 9177 full-length reference transcripts out of 43757 candidates. Again Trinity gets more false positives than BinPacker and Bridger. The reference true positive rate of BinPacker is 14.05%, best of all *de novo* assemblers, while that of Trinity is 11.27%, higher than all the other assemblers except Bridger, which performs only a little worse than BinPacker in this measure, with its reference true positive rate 14.03%; the assembled true positive rate of BinPacker achieves 10.37%, while Trinity reaches 11.14%, highest among the compared *de novo* assemblers, including Bridger, with its assembled true positive rate 9.56%. We also compute the sensitivity and accuracy distributions against recovered sequence length rates ranging from 80% to 100%. For the sensitivity distribution, the three curves of BinPacker, Bridger and Trinity are almost coincident with the highest sensitivity among all *de novo* assemblers. For the accuracy, the reference true positive rate of BinPacker keeps the highest in the interval [90%, 100%]. For the assembled true positive rate, BinPacker performs a little worse than Trinity, which reaches the highest in the whole interval, but much better than the others. The performance of the other assemblers on human dataset is almost the same as on dog and mouse datasets (See Figs 1B–3B for details).

### 2. Tests on simulated dataset

It is necessary to test the assemblers using simulated RNA-seq dataset since we may know all the genuine transcripts hidden in it in advance. An *in silico* RNA-Seq data generator, Flux Simulator [22], is applied to UCSC hg19 gene annotation to generate an error-free dataset of approximately 50 million paired-end strand-specific RNA-seq reads. To demonstrate the advantage of BinPacker over other assemblers on the simulated dataset, we ran all the assemblers and did comparison among them in terms of their sensitivities, accuracies and their distributions against recovered sequence length rates.

**Fig 4:**
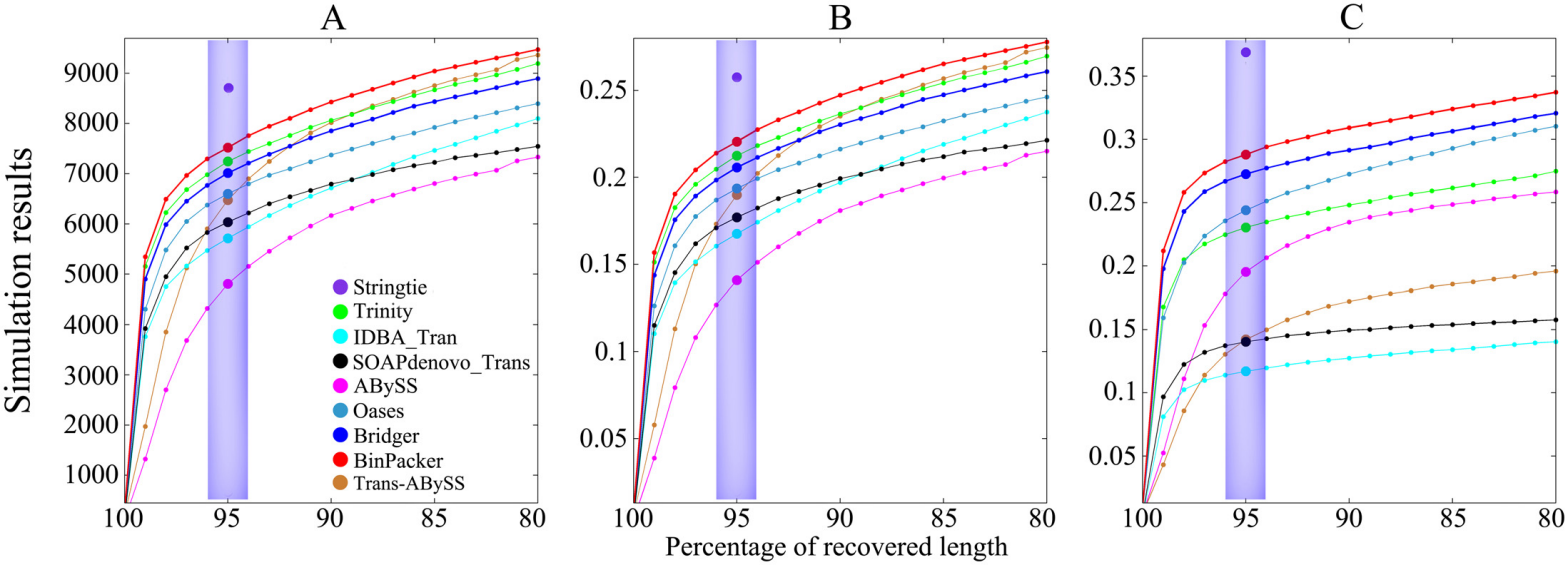
Comparison of assemblers on simulated dataset. (A) Recovered reference sensitivity and its distribution against recovered sequence length rates. The solid colored circles in shaded areas represent the number of full-length recovered reference transcripts for different assemblers; (B) Reference true positive rate and its distribution against recovered sequence length rates. The solid colored circles in shaded areas represent the reference true positive rate for different assemblers; (C) Assembled true positive rate and its distribution against recovered sequence length rates. The solid colored circles in shaded areas represent the assembled true positive rate for different assemblers.

Our comparison results show that BinPacker not only reaches the highest sensitivity, but also the highest accuracy levels of both types. Furthermore, BinPacker keeps the highest sensitivity and accuracy of both types in the whole interval [80%, 100%]. Therefore it can be concluded that BinPacker has the highest reliability among all the *de novo* assemblers we are comparing with in terms of their distributions of both sensitivity and accuracy against recovered sequence length rates on the simulated dataset. See Fig 4 and S1 Text for details.

### 3. Comparison of running time and memory usage on real datasets

**Fig 5:**
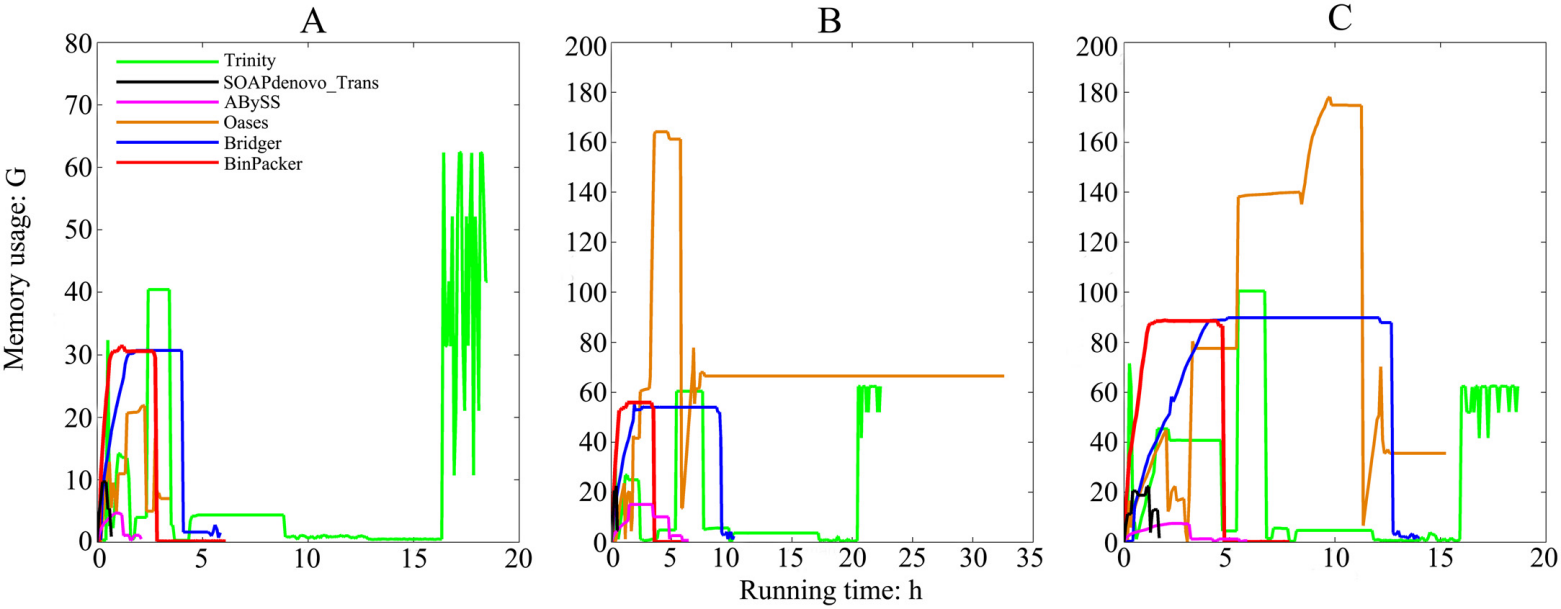
RAM usage for each assembler on (A) dog, (B) human and (C) mouse datasets. Same parameter values are used for all assemblers: k = 25 and CPU = 6.

**Fig 6:**
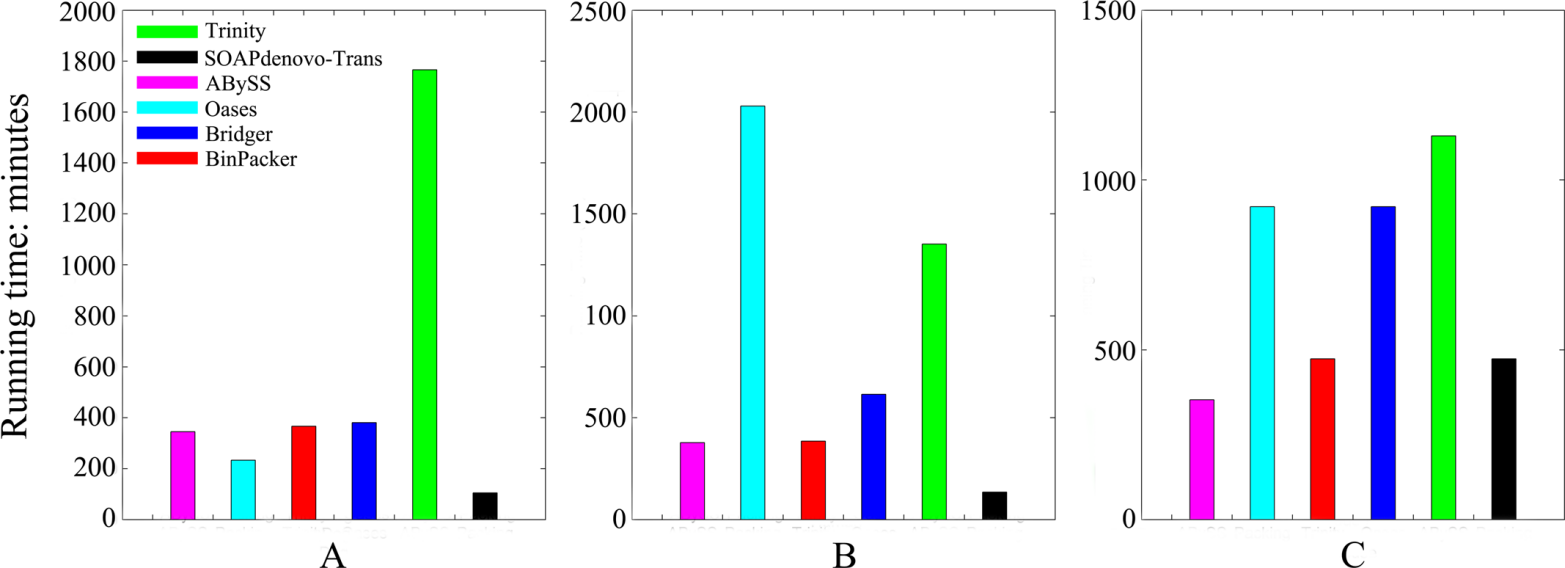
Running time for each assembler on (A) dog, (B) human and (C) mouse datasets.

We examined the computing resources required by these *de novo* assemblers: the running time and the memory usage on the same server. The results are shown in Figs 5 and 6. ABySS uses the least memory (Fig 5), while SOAPdenovo-Trans takes the shortest time (Fig 6). Oases performs well on dog dataset but it consumes the most memory and has almost the longest running time on both human and mouse dataset. We noted that the computing resource requirement by Oases is sensitive to the *k*-mer value, which had also been found in another research paper [23]. As an enumeration algorithm, Trinity consumes the most memory on dog dataset and takes the longest time on both dog and mouse datasets. For the memory usage (Fig 5) BinPacker and Bridger almost require the same amount of memory, more than most of the compared assemblers except Trinity and Oases, which consume much more memory than BinPacker on human and mouse datasets. For the time usage (Fig 6), BinPacker is among the fastest assemblers and it has also made a great improvement compared to Bridger, which takes much more time than BinPacker on both human and mouse datasets.

## Discussion

In this study, we presented a novel *de novo* method BinPacker for transcriptome assembly using short RNA-seq reads. Compared with Trinity, one of the most popular *de novo* assemblers, BinPacker has the following advantages: (i) Trinity uses a fixed k-mer length 25, which is not necessarily optimal for all datasets, while BinPacker allows different user-specified k-mer values for different problems for optimal performance. One crucial parameter of BinPacker is the k-mer length. Generally speaking, with larger k values it performs better on high expression datasets or longer reads and with smaller k values it performs better on low expression datasets or shorter reads [17]. In light of our testing results, k = 25 is chosen to be the default value, however, larger k values are recommended for reads with length longer than 75bp. (ii) Compared to the exhaustive enumeration method used in Trinity, BinPacker uses a rigorous mathematical model to search for an optimal set of paths from the splicing graph, which makes BinPacker achieve a lower false positive rate at the same level of sensitivity. (iii) BinPacker makes full use of the sequencing depth information, which is applied to define the junction weights of the splicing graphs, constraining the deconvolution of splicing graphs into individual transcripts, and hence making its assembly results more accurate. (iv) BinPacker makes a different use of the paired-end information compared with Trinity. While Trinity uses the paired-end information to search for paths in the *de Bruijn* graphs, this information is mainly used in our process of constructing splicing graphs. Firstly, the paired-end information is used to help reconstruct more complete splicing graphs, making contigs even not covered by overlapping k-mers be recovered during assembly. Secondly, paired-end information is also used to trim error branches of the constructed splicing graphs, removing error junctions from splicing graphs. In practice, BinPacker uses less memory space and shorter running time.

As showed in Results section, the assemblers have a high variance in sensitivity, accuracy and time and memory usage across the different RNA-seq datasets. Several facts may cause such a variance. 1) Different RNA-Seq datasets may contain different transcripts expression levels and different sequencing depths, both of which lead to the same transcripts in different RNA-Seq datasets covered by quite a different number of reads. And so they could have a large effect on sensitivity, accuracy and time and memory usage. 2) The reads in different RNA-Seq datasets may have different lengths, maybe shorter than 50, and maybe longer than 100, which may also cause differences in sensitivity, accuracy and time and memory usage. 3) The qualities of reference transcripts for different species are also quite different. For example, human and mouse genomes have been studied more extensively than dog genome, so the rate of known reference transcripts will certainly be larger than that of dog. We have seen in our comparison the sensitivity and accuracy of dog is lower than that of human and mouse. 4) Other reasons, such as different sequencing error rates, the usage of paired-end reads or single-end reads, may also contribute to the variance in sensitivity, accuracy and time and memory usage.

The E. coli dataset is also adopted to evaluate the performance of the *de novo* assemblers on low complexity genome species without alternative splicing isoforms. Since the dataset is much smaller than that of dog, human and mouse, all the compared assemblers use much less running time and memory usage. For the sensitivity and accuracy, because most compared assemblers are designed to assemble transcripts from genes with alternative splicing events, they all do not perform very well on low complexity genome species such as E. coli without alternative splicing isoforms. Details are described in the first section of the S1 Text.

As far as we know, BinPacker is the first algorithm using the bin-packing strategy for *de novo* assembly, without the utilization of any other reference information. Tested on both real and simulated RNA-seq datasets, BinPacker shows the best sensitivity and accuracy compared to all the other *de novo* assemblers, and even outperforms the most popular *ab initio* assembler StringTie on real dog dataset, only slightly worse than Trinity in some aspects on real human dataset. In addition, it requires fewer computational resources and less running time compared to most of the other assemblers. With these demonstrated advantages, we anticipate that BinPacker will play an important role for new discoveries in transcriptome study using RNA-seq datasets, especially for cancer transcriptomic data analyses.

## Methods

The splicing graph was first introduced by Heber, et al in 2002 [24]. BinPacker assembles transcripts on each splicing graph it constructs. Each splicing graph constructed by BinPacker is a directed acyclic graph, with its nodes and edges representing exons and splicing events of the gene. The nodes in the splicing graph are continuous genome sequences without any alternative splicing events, which may not be real exons of the gene. Based on the generalized definition of exons, BinPacker first builds splicing graphs for all expressed genes encoded in the genome using the given RNA-seq datasets. Ideally, each splicing graph constructed by BinPacker has a correspondence to a specific (expressed) gene. Unfortunately, it may not always be this case due to the existence of sequencing errors, homologous genes and low expression levels of some genes. But it does not cause a serious impact on our full-length transcripts recovery of individual genes even though some splicing graphs cover multiple genes or only parts of a gene. Without loss of generality, we assume that each splicing graph represents one expressed gene. Having constructed all the splicing graphs, BinPacker searches for an optimal edge-path-cover over all the individual splicing graphs by iteratively solving a series of bin packing problems. Each edge-path-cover output by BinPacker can explain all the observed splicing events encoded in the corresponding splicing graph. A flowchart of the BinPacker algorithm is given in Fig C in S1 Text.

### 1. Construction of splicing graphs

BinPacker constructs splicing graphs based on the method of Bridger [17] with several updates as follows. First of all, while Bridger is not able to process RNA-Seq reads with different lengths, BinPacker can handle reads with variable lengths. Secondly, Bridger trims the branches of the splicing graphs after all splicing graphs have been constructed. However, BinPacker trims splicing graphs during the construction of splicing graphs.

### 2. Topological ordering of splicing graph and detecting a maximal set of pairwise incompatible edges

Two directed edges in a splicing graph are said to be compatible if they may come from one directed path, and incompatible otherwise (see Fig I in S1 Text). We may imagine that the splicing graphs one-to-one correspond to the expressed genes, with nodes corresponding to exons and edges corresponding to splicing junctions. Since exons are linearly arranged in a gene, we may suppose that the nodes in the splicing graph of the gene are also linearly arranged, but not necessarily to be identical to that of the gene. We did this linearly arrangement by topological ordering of the splicing graph, which can be solved in linear time [25]. After topological ordering, all nodes with only out-edges are moved to the leftmost of the graph and all nodes with only in-edges to the rightmost. From now on, we refer to the splicing graph with all nodes being linearly arranged as a canonical splicing graph. Note that each directed edge in the canonical splicing graph can only go in the direction of the gene (Fig I in S1 Text). Each edge in a splicing graph is assigned a weight using its sequencing depth (number of reads spanning the junction edge in the splicing graph). It is obvious that the edges crossing two consecutive nodes in the splicing graph are pairwise incompatible (Fig J in S1 Text). In fact, the maximum set of edges crossing two consecutive nodes in a canonical splicing graph must be a maximal set of pairwise incompatible edges in the splicing graph (see Theorem 1 in S1 Text). BinPacker will iteratively execute a series of bin packing programs based on such a maximal set of pairwise incompatible edges.

### 3. Bin packing

BinPacker iteratively calls a variant of bin packing model to comb all the transcripts encoded in a splicing graph. To do so, we add a source node *s* and a sink node *t* into the splicing graph (Fig I in S1 Text), and connect *s* to the nodes with only out-going edges, and connect all the nodes with only in-coming edges to *t*. The weight of the new edge connecting *s* and *u* is assigned to be the sum of the weights of the edges going out from *u*. Similarly, the new edges going to *t* can be weighted.

#### Step 1: Balancing splicing graphs

Let *u* be a node in a splicing graph, the sum of the weights of the in-edges of *u* is said to be in-weight of *u*, denoted by *w*_*in*_(*u*). Out-weight of *u* is defined similarly, denoted by *w*_*out*_(*u*). Let *w*_*min*_ = min{*w*_*in*_(*u*), *w*_*out*_(*u*)}, *c* = *α*(*γ*—*β*)/*w*_*min*_ *+ β*, where *α*, *β* and *γ* are parameters that users can specify (see Methods and Fig M in S1 Text for details). When there is a significant difference between *w*_*in*_(*u*) and *w*_*out*_(*u*), the node u is supposed to be an end of a transcript. We handle this by adding a new edge from the source s to the node u, with weight *w*_*out*_(*u*)*−w*_*in*_(*u*) whenever *w*_*out*_*(u)/w*_*in*_*(u) ≥ c*, which means that the difference between *win*(*u*) and *w*_*out*_(*u*) is significant. Similarly, we may add a new edge from the node u to the sink t if *w*_*in*_(*u*)*/w*_*out*_(*u*) *≥ c*. BinPacker sets α = 10, β = 1.4 and γ = 1.5 as default.

#### Step 2: Iterations of the bin packing

Suppose that we have a maximal set *I* of pairwise incompatible edges crossing the two consecutive nodes obtained above. BinPacker identifies each edge in the splicing graph as a bin with its capacity being the weight (sequencing depth) of the edge, and puts an item *i* in each bin (edge) in *I*. The size of the item *i*, denoted by *w*_*i*_, is simply the weight of the edge (bin) where the item *i* resides. During the execution, BinPacker always faces a bin packing problem, which is slightly different from the traditional bin packing model. In our model, each item must be packed into one and only one bin and each bin can hold several items with the sum of their sizes smaller or larger than or equal to the capacity of the bin. At the very beginning, all the |*I*| items are one-to-one put in the |*I*| bins accordingly. Let *n*_*L*_ denote the left one of the two consecutive nodes and *n*_*R*_ the right one.

**Fig 7:**
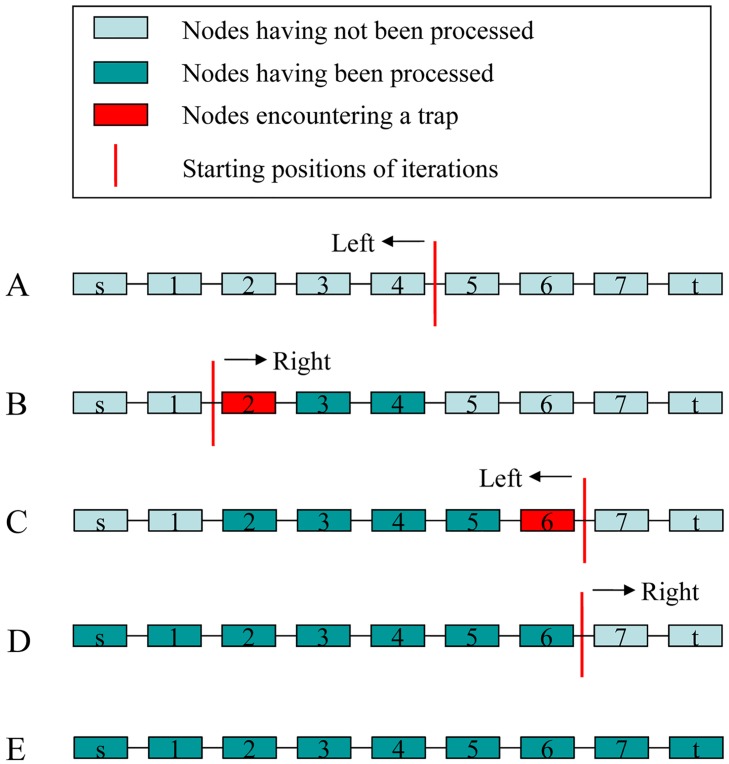
An example which shows the iterations of BinPacker in the packing process. (A) The first iteration of BinPacker starts from node 4 to the left. (B) When BinPacker processes nodes one by one to the left, it encounters a trap node 2, and then enters the next iteration starting from node 2 to the right. (C) When BinPacker processes nodes to the right, it encounters another trap node 6 and then enters the next iteration starting from node 6 towards left. (D) In this iteration, BinPacker reaches a terminal *s*, and then jumps back to the starting node 6 and processes the remaining nodes one by one to the right. (E) BinPacker reaches another terminal *t* and terminates its iterations.

Starting from *n*_*L*_, BinPacker carries out the first iteration of the bin packing. The first instance of bin packing towards left is formed as follows: we have as input the |*I*| items defined from the edges in *I*, and a set *I'* of bins (edges) crossing the two consecutive nodes *n*_*L*_*-1* and *n*_*L*_. What we are going to do is to optimally pack the |*I*| items into the |*I'*| bins. For the (heuristic) algorithm design, we partition the edges in *I* U *I'* into three sets *I*_*in*_, *I*_*out*_ and *I*_*m*_, with *I*_*in*_ consisting of the edges coming to *n*_*L*_, *I*_*out*_ of the edges going out of *n*_*L*_, and *I*_*m*_ of the remaining edges. Clearly, edges in *I*_*in*_ belong to *I'* but not to *I*, edges in *I*_*out*_ belong to *I* but not to *I'*, and edges in *I*_*m*_ belong to both *I* and *I'*. For the first instance, we have that *|I*_*out*_*|≥|I*_*in*_*|* since *|I|≥|I'|*. Executing the bin packing, BinPacker keeps the items in the bins (edges) of *I*_*m*_ staying unchanged, and optimally packs the items in the bins (edges) of *I*_*out*_ into the bins (edges) of *I*_*in*_ whenever *|I*_*out*_*|≥|I*_*in*_*|*, then reset *n*_*L*_ *= n*_*L*_*-1*, *I*_*in*_ and *I*_*out*_ accordingly. Repeat this procedure until encountering a *trap node n*_*L*_ with *|I*_*out*_*|<|I*_*in*_*|*, which may happen in two cases (see Methods in S1 Text), or reaching the source node *s*. If the former occurs in some iteration, BinPacker replaces the *n* (= |*I*_*out*_|) items in the bins (edges) of *I*_*out*_ by *m* (= |*I*_*in*_|) new items with sizes, *w*_*1*_, *w*_*2*_,…, *w*_*m*_, the weights of the *m* edges of *I*_*in*_, while the other items in the bins (edges) of *I*_*m*_ stay unchanged, and then executes the next iteration starting from the trap node *n*_*L*_ towards the opposite direction until encountering another trap node or reaching the sink node *t*; otherwise, the latter occurs, BinPacker jumps back to the starting node of the current iteration and processes the remaining nodes one by one until encountering a new trap node or reaching the sink node *t*. Repeat the procedure until all nodes are processed (see Fig 7). The forth and back iteration must terminate within a few times (no more than |*V*| times, where *V* is the node set of the splicing graph) due to the fact that the nodes previously processed will never be trapped again (see Theorem 2 in S1 Text).

#### Step 3: Bin packing by 0–1 quadratic programming

Suppose that BinPacker is processing the node *n*_*L*_ towards left and we have *m* edges (bins) coming to *n*_*L*_ from which the bins (edges) containing *n* items go out.

If *m*≤*n*, the bin packing can be solved by the following 0–1 quadratic programming:

$$\begin{array}{l} \text{min}f=\sum_{i=1}^{m}{\left(c_{i}-\sum_{j=1}^{n}w_{j}x_{ij}\right)}^{2}\\ \begin{array}{cc} s.t. & \{\begin{array}{c} \begin{array}{cc} \sum_{i=1}^{m}x_{ij}=1 & \forall j=1,\ldots ,n \end{array}\\ \begin{array}{cc} \sum_{j=1}^{n}x_{ij}\geq 1 & \forall i=1,\ldots ,m \end{array}\\ x_{ij}\in \{0,1\} \end{array} \end{array} \end{array}$$ (1)

where *c*_*i*_ represents the capacity of bin *i*, *w*_j_ the size of item *j*, and *x*_*ij*_ is a binary variable with *x*_*ij*_ = 1 if item *j* is packed into bin *i*, 0 otherwise. The first constraint ensures that one item goes into one and only one bin, and the second one guarantees that each bin receives at least one item.

Otherwise, we have *m*>*n*, and get trapped at the node *n*_*L*_. Then the bin packing can be solved by the following quadratic programming:

$$\begin{array}{l} \text{min}f=\sum_{i=1}^{\text{k}}{\left(c_{i}-\sum_{j=1}^{m}w_{j}x_{ij}\right)}^{2}\\ \begin{array}{cc} s.t. & \{\begin{array}{c} \begin{array}{cc} \sum_{i=1}^{\text{k}}x_{ij}=1 & \forall j=1,\ldots ,m \end{array}\\ \begin{array}{cc} \sum_{j=1}^{m}x_{ij}\geq n_{i} & \forall i=1,\ldots ,\text{k} \end{array}\\ x_{ij}\in \{0,1\} \end{array} \end{array} \end{array}$$ (2)

where *k* represents the number of edges going out of *n*_*L*_, *n*_*i*_ the number of items packed into bin (edge) *i* (1 ≤ *i* ≤ *k*). This constraint ensures that each bin previously packed will get at least the same number of items as in the last iteration.

#### Step 4: Transformation into 0–1 ILP

The 0–1 quadratic programming can be equivalently transformed into a 0–1 linear programming (see Methods and Theorem 3 in S1 Text for details). To do so, we simply introduce a new variable *x*_*ijik*_ for each quadratic term *x*_*ij*_*·x*_*ik*_ (or *x*_*ik*_*·x*_*ij*_) in the objective function along with the constraints:

$$\{\begin{array}{c} \begin{array}{ccc} x_{ijik}\leq x_{ij} & \forall i=1,\ldots ,m & 1\leq j\leq k\leq n \end{array}\\ \begin{array}{ccc} x_{ijik}\leq x_{ik} & \forall i=1,\ldots ,m & 1\leq j\leq k\leq n \end{array}\\ \begin{array}{ccc} x_{ij}+x_{ik}-1\leq x_{ijik} & \forall i=1,\ldots ,m & 1\leq j\leq k\leq n \end{array}\\ \begin{array}{ccc} x_{ijik}\in \{0,1\} & \forall i=1,\ldots ,m & 1\leq j\leq k\leq n \end{array} \end{array}$$

### 4. Recovery of an optimal set of full-length transcripts

All the 0–1 ILPs are optimally solved by GLPK-4.40. The GLPK (GNU Linear Programming Kit) package is intended for solving large-scale linear programming (LP), mixed integer programming (MIP), and other related problems. It is a set of routines written in ANSIC and organized in the form of a callable library. Since each programming is modeled from one node of a splicing graph, the number of variables of the 0–1 ILP is |*I*_*in*_|⋅|*I*_*out*_|⋅(|*I*_*out*_|+3)/2 or |*I*_*out*_|⋅|*I*_*in*_|⋅(|*I*_*in*_|+3)/2. In most cases, |*I*_*out*_|<3 and |*I*_*in*_|<3, so the number of variables of the 0–1 ILP is almost always less than 27 and it can be optimally solved by GLPK extremely fast. And even in many cases, |*I*_*out*_| = 1 or |*I*_*in*_| = 1, in which cases, items can be directly packed into corresponding bins without using GLPK.

The solution {*x*_*ij*_} tells us that item *j* is in bin *i* if and only if *x*_*ij*_ = 1. All the bins (edges) containing the same item induce an *s-t* path in the splicing graph of a gene which may correspond to a transcript of the gene. Finally, BinPacker outputs all the transcripts induced by individual items in the splicing graph of the gene.

## Supporting Information

S1 Text Supplementary material of BinPacker. (PDF)
