# Supplementary material for: BinPacker: Packing-Based De Novo Transcriptome Assembly from RNA-seq Data
Source: PLoS Comput Biol. 2016 Feb 19;12(2):e1004772. doi: 10.1371/journal.pcbi.1004772 (PMC4760927; doi:10.1371/journal.pcbi.1004772)
Supplement: S1 Text — (PDF) [file pcbi.1004772.s001.pdf]

Supplementary Material for the Paper

BinPacker: Packing-based *de novo* Transcriptome Assembly from RNA-seq Data

Juntao Liu[1, †], Guojun Li[1,†,*], Zheng Chang[1], Bingqiang Liu[1], Rick McMullen[3], Pengyin Chen[4], Xiuzhen Huang[2, *]

1. Supplementary Notes: parameter setup for the compared assemblers

1.1 Simulated and E.coli datasets

Parameters are set up as follows for each algorithm: ABySS: "abyss-pe k=25 c=2 E=0 j=6 in = 'left.fq right.fq' "; Trans-ABySS was run by setting k-mer length k to 25, 29, 33, 37, 43; Trinity: "--CPU 6 --bflyHeapSpaceMax 10G --bflyGCThreads 4 --SS_lib_type RF"; BinPacker: "k=29 --SS_lib_type RF"; Oases: "-ins_length 200 -cov_cutoff 2 -edgeFractionCutoff 0.05 -min_trans_lgth 200"; SOAPdenovo-Trans was run using default parameters; IDBA-Tran: "-mink 21 -maxk 37 -step 4"; Stringtie was run using default parameters; Bridger: "k=25 --SS_lib_type RF". All the assemblers were tested on a server with 512GB of RAM.

1.2 Real datasets

Parameters are set up as follows for each algorithm: ABySS: "abyss-pe k=25 c=2 E=0 j=6 in = 'left.fq right.fq' "; Trans-ABySS was run by setting k-mer length k to 25, 29, 33, 37, 43; Trinity: "--CPU 6 --bflyHeapSpaceMax 10G --bflyGCThreads 4" for non-strand specific human and dog data and "--SS_lib_type RF" for the strand-specific mouse data; BinPacker: k=25 for dog and human data while k=31 and "--SS_lib_type RF" for mouse data; Oases: "-ins_length 200 -cov_cutoff 2 -edgeFractionCutoff 0.05 -min_trans_lgth 200"; SOAPdenovo-Trans was run using default parameters; IDBA-Tran: "-mink 21 -maxk 43 -step 4" for human and dog data and "-mink 21 -maxk 37 -step 4" for mouse data; Stringtie was run using default parameters; Bridger: k=25 for dog and human data while k=31 and '--SS_lib_type RF' for mouse data. All the assemblers were performed on a server with 512GB of RAM.

2. Supplementary Methods

BinPacker constructs splicing graphs effectively and efficiently

A splicing graph of a gene is a directed acyclic graph, whose nodes represent exons and two nodes are connected by an edge if and only if some splicing event occurs between them. Several splicing events and their corresponding splicing graphs are shown in Figure D. It is worthy of mentioning that while BinPacker is initially developed to handle exon skip, the main alternative splicing events in mammalian transcriptomes, the same framework can deal with general splicing events including intron retention and alternative splicing sites as demonstrated in Figure D(ii) and D(iii). The key is that we introduced the concept of artificial exon, which is a continuous genome sequence without any alternative splicing events.

Basically, based on this generalized definition of exon, all alternative splicing events could be handled using the algorithm below.

BinPacker builds splicing graphs for all genes encoded in the genome based on the given RNA-seq data. In an ideal situation, the constructed graphs would have a one-to-one correspondence to all the (expressed) genes. However, it is not always the case due to homologous genes and low sequence coverage for some genes. In spite of this, it will not lead to a serious problem for our ultimate recovery of full-length transcripts of individual genes even if some splicing graphs may cover multiple genes or only parts of a gene. Hence, we may assume that each constructed splicing graph represents RNA-seq data from one single gene.

We first build a hash table from all the reads. For each *k*-mer (default $k = 25$) occurring in the reads, the hash table records the abundance of that *k*-mer and the IDs of reads containing that *k*-mer. To reducing memory usage, each *k*-mer is stored as a 64-bit unsigned integer with 2-bit nucleotide encoding, thus the parameter *k* is not allowed to be larger than 32. Then, we remove error-containing *k*-mers and select seed *k*-mers by the same strategy in Trinity [1]. A *k*-mer is chosen as a seed must meet the following criteria: (a) Shannon's Entropy [2] of the *k*-mer $H \geq 1.5$, (b) the *k*-mer occurs at least twice in the complete set of input reads, and (c) the *k*-mer is not palindromic[1]. The seed *k*-mer is extended to a complete splicing graph greedily in the following steps:

(1) We extend the seed *k*-mer in two directions by repeatedly selecting the most frequent *k*-mer in the hash table, overlapping *k*-1 nucleotides with the current contig terminus, in order to provide a single-base extension. A *k*-mer will be immediately marked as long as it was used in the construction of current splicing graph and the marked *k*-mer will not be used again in this graph. However, during the construction of splicing graphs, a marked *k*-mer is allowed to be used for the extension of a splicing graph if and only if the marked *k*-mer has not been in the graph and the graph cannot be extended without the marked *k*-mer. But a marked *k*-mer is not allowed to be selected as a new seed for the construction of next splicing graphs.

(2) When the contig cannot be extended, we use paired-read information to get further extension. Based on our hash table, the reads mapping to the terminus of this contig are easily collected. If some of their paired-end reads are not used in the current splicing graph, it implies that the contig is not complete. We may generate a new contig from an unused paired-read, and then connect it to the existing contig by using the pair information (Figure E). Thus, some transcripts that cannot be covered by overlapping *k*-mers would be reconstructed. The ultimate contig is used as the trunk of a splicing graph to be constructed.

(3) We check each *k*-mer in the trunk to see if there exists a *k*-mer having an alternative extension that has not been used (such a *k*-mer is called a bifurcation *k*-mer). Once a bifurcation *k*-mer is found, we extend it in the same way as above.

(4) During the extension in step (3), if the current branch can be extended by some used *k*-mer in the current splicing graph, we identify a new bifurcation *k*-mer and modify current splicing graph by merging k-1 overlapping nucleotides and adding one directed edge between them (Figure F). Otherwise, the following criteria are used to check if this potential branch is allowed to add to current splicing graph: (i) the branch is long enough (>=80 bp) to be an exon (Figure G(i)); (ii) the branch is not similar with corresponding part of the trunk (Figure G(ii)); (iii) there are at least two read pairs supporting this branch (Figure G(iii)).

Two paralogous genes can be separated by using paired-end read information (Figure G(iv)). The new branch will not be added into current graph if we find a paired-end read with one end (colored green) mapping to the branch and the other end mapping to outside of the current graph (note that the red dash does not exist in current splicing graph). When we construct the splicing graph of the red gene, the "hole" resulting from the first gene (black gene) can be filled by used *k*-mers (The used *k*-mers are only allowed to be reused to fill such holes).

(5) We grow the splicing graph by repeatedly finding the bifurcation *k*-mers, until no bifurcation *k*-mer exists.

(6) We mark all used *k*-mers and trim edges induced from sequencing errors by the similar criteria used in Trinity: (a) for each edge, there is a minimal number of reads (default 2) perfectly match at least (*k*-1)/2 bases on each side of the junction. (b) The coverage of each edge must exceed 0.04 times the average coverage of two franking nodes (twice the sequencing error rate in a read, the upper bound is about 2%). (c) If there is a node with several outgoing edges, each one of them should have a read support more than 5% of the total outgoing reads. (d) Any outgoing edge has a support more than 2% of the total incoming reads. Edges in splicing graph that does not meet any one of these criteria are removed.

Splicing graphs with less than minimum number of *k*-mers are discarded (an empirical value used by Trinity is 300-(k-1) =276). For non-strand specific RNA-seq data, both *k*-mer and the reverse-complemented *k*-mer are considered in building the hash table, extending the splicing graph. A splicing graph is a compacted directed acyclic graph, and ideally each node, which is a fragment of sequence, corresponds to one exon and each edge represents one junction.

Note that the splicing graphs constructed here cannot be obtained by a contracted *de Bruijn* graph. One example showing their differences can be found in Figure H. In addition, the *de Bruijn* graph usually suffers a problem that the first graph built from the hash table is very huge because many genes are mixed together by sharing their *k*-mers. However, the splicing graphs constructed here could keep their size smaller by using paired-end read information to check if a new branch should be added, which makes finding transcripts from the graph much easier.

Splicing graphs provide a natural and lossless representation of all the (alternatively) splicing isoforms in a transcriptome, with each node corresponding to one exon and each edge representing one splicing junction, where a splicing event between two exons takes place. By analyzing the structure of splicing graphs, we discovered that full-length transcripts would be better recovered from combinations of spliced junctions than of exons and that the transcriptome of a gene would be better identified as a constrained minimum edge-path-cover over the splicing graph than simply a minimum edge-path-cover. Thus we model the *de novo* assembly problem as to find a constrained minimum edge-path-cover over a splicing graph, in which way full-length transcripts would be precisely recovered almost without any loss of the (alternatively) splicing isoforms in the transcriptome.

BinPacker sorts nodes of a splicing graph and detects a maximal set of pairwise incompatible edges

Two directed edges in a splicing graph are said to be compatible if they may come from one directed path, and incompatible otherwise. As shown in Figure I, edges 1 and 2 may clearly come from the same directed path, so they are compatible. For edges 1 and 3, they may be in the path: node 1→node 3→node 4→node 6, so they are also compatible. While for edge 2 and edge 3, there is no directed path passing both of them, so they are incompatible. We may imagine that the splicing graphs one-to-one correspond to the expressed genes, with nodes corresponding to exons and edges corresponding to splicing junctions. Since exons are linearly arranged in a gene we may suppose that the nodes in the splicing graph of the gene are all arranged linearly by topology ordering of the nodes of the splicing graph, but not necessarily identical to the gene. After topology ordering, all nodes with only out-edges are moved to the leftmost of the graph and all nodes with only in-edges to the rightmost.

From now on, we call a splicing graph arranged up by the algorithm above a canonical splicing graph. Therefore, each directed edge in a canonical splicing graph goes in the direction of the gene to which the splicing graph corresponds and each edge is assigned to a weight by the sequencing depth (number of reads spanning the junction edge in the splicing graph) of the junction to which the edge corresponds. A directed edge e is said to cross two consecutive nodes n_i and n_{i+1} if its tail is in the left of node n_i (including node n_i) and its head is in the right of node n_{i+1} (including node n_{i+1}). As shown in Figure J, the edges 1, 2 and 3 all cross the two consecutive nodes 3 and 4. Obviously, edges crossing two consecutive nodes must be pairwise incompatible. Two edges being incompatible means that they are not reachable from each other.

Theorem 1 The maximum set of edges crossing two consecutive nodes in a canonical splicing graph must be a maximal set of pairwise incompatible edges.

Proof Let I be a maximum set of edges crossing the two consecutive nodes n_i and n_{i+1}. Assume to the contrary that I is not a maximal set of pairwise incompatible edges. Then there

is an edge e which is not in I and not reachable from any edges in I. Assume without loss of generality that the edge e locates at the right site of n_{i+1}, *i.e.*, $e=(n_s, n_t)$ with $s \geq i+1$. The unreachability of e from I implies that the node n_s has no in-edges. It follows from the construction of a canonical splicing graph that all the nodes before n_s have no in-edges. Therefore all the edges in I have to pass the consecutive nodes n_s and n_{s+1}, a contradiction to the hypothesis that I is a maximum set of edges crossing two consecutive nodes in the canonical splicing graph. □

BinPacker executes bin packing

BinPacker iteratively calls a variant of bin packing model to comb all the transcripts encoded in a splicing graph. To do so, we add a source node s and a sink node t into the splicing graph, and connect s to the nodes with only out-going edges, and connect all the nodes with only in-coming edges to t. The weight of the new edge connecting s and u is defined to be the sum of the weights of the edges going out from u. Similarly, the new edges going to t can be weighted.

Step 1. Balancing splicing graphs. Let u be a node in a splicing graph, and the sum of the weights of the in-edges of u is said to be in-weight of u, denoted by $w_{in}(u)$. Out-weight of u is defined similarly, denoted by $w_{out}(u)$. Due to the existence of noises to the RNA-seq data, it is difficult to have an exact balance between $w_{in}(u)$ and $w_{out}(u)$ for any node u. When the difference between $w_{in}(u)$ and $w_{out}(u)$ is relatively small, it is rationale for us to believe that the difference would be caused by noises. However, if the difference between $w_{in}(u)$ and $w_{out}(u)$ is significant, we would prefer that the node u is supposed to be an end of a transcript. The problem is how big the difference between $w_{in}(u)$ and $w_{out}(u)$ is qualified to be significant? Here we provide concrete examples to demonstrate that the significance of difference between $w_{in}(u)$ and $w_{out}(u)$ relies on the magnitude of both $w_{in}(u)$ and $w_{out}(u)$. As an example where $w_{in}(u) = 1$ and $w_{out}(u) = 11$ with their difference being 10, it is rationale for us to say that the difference between $w_{in}(u)$ and $w_{out}(u)$ is significant. However, we cannot say anything if $w_{in}(u) = 1000$ and $w_{out}(u) = 1010$ even though the difference is also 10. We further tried to measure it using the ratio $w_{out}(u)/w_{in}(u)$ (or $w_{in}(u)/w_{out}(u)$), and found that it does not work either. For example, both $w_{in}(u) = 1000$, $w_{out}(u) = 1500$ and $w_{in}(u) = 2$, $w_{out}(u) = 3$ have their ratio 1.5, but then we would prefer the former having a significant difference other than the latter. Therefore, only the difference or ratio between $w_{in}(u)$ and $w_{out}(u)$ is not enough to guarantee whether or not the node u would be an end of a transcript.

The above observations imply that the significance of the difference between $w_{in}(u)$ and $w_{out}(u)$ depends on both their ratio and the minimum one of them. The smaller $w_{min} = min\{w_{in}(u), w_{out}(u)\}$ is, the bigger we need the ratio $w_{out}(u)/w_{in}(u)$ (or $w_{in}(u)/w_{out}(u)$) to be to guarantee the significance. So we define a threshold c as an inverse proportional function $c=k/w_{min}+b$ of w_{min} such that the node u is expected to be an end of some transcript only if

either $w_{out}(u)/w_{in}(u) \geq c$ or $w_{in}(u)/w_{out}(u) \geq c$. We also call such a node to be significant. For each significant node u, we add a new edge with weight $w_{out}(u)$-$w_{in}(u)$ from the source s to the node u if $w_{out}(u)/w_{in}(u) \geq c$, or a new edge with weight $w_{in}(u)$-$w_{out}(u)$ from the node u to the sink t if $w_{in}(u)/w_{out}(u) \geq c$.

To determine the parameters k and b, we should set the asymptote $y=\beta$ and specify a point (α, γ) on the function curve. The asymptote $y=\beta$ means that the ratio $w_{out}(u)/w_{in}(u)$ (or $w_{in}(u)/w_{out}(u)$) can never be lower than β if node u is significant. The point (α, γ) on the function curve indicates that if w_{min} is α then the ratio $w_{out}(u)/w_{in}(u)$ (or $w_{in}(u)/w_{out}(u)$) is at least γ to ensure that node u is significant. After the parameters α, β and γ are specified, we have that $c=\alpha(\gamma-\beta)/w_{min}+\beta$.

To set default values for α, β and γ, we optimize them by cross-validation strategy on simulated RNA-seq datasets with different numbers of reads and different lengths of reads. The final selected values are α=10, β=1.4 and γ=1.5. To test the robustness of these parameters, we conducted two experiments on both real and simulated datasets. In each experiment, we did some variations on these parameters and compute their sensitivity, assembled true positive rate and reference true positive rate, respectively. Figure K shows the results for real mouse data and Figure L for the simulated human data. We are convinced from the results that the default values for the three parameters α, β and γ are reasonable for different RNA-seq datasets because they show significant robustness around the default values. Figure M shows the implementation of the parameters.

Step 2. Iterations of the bin packing. Iteration details have been presented in the main article, where it mentions that a *trap node* may occur in two cases as follows. i) The maximal set I of pairwise incompatible edges is not maximum. As shown in Figure N(i), the set I contains edges 3, 4 and 5, crossing the two consecutive nodes 4 and 5. Obviously, I is not maximum because the edges 1, 2, 3 and 4, colored purple in Figure N(i), form the maximum set of pairwise incompatible edges. When BinPacker processes node 3, it encounters the case $m > n$ (m = 2 and n = 1). ii) Locally seeking the optimality, BinPacker may encounter the situation of $m > n$ because it tends to pack more items into a bin with larger capacity as well as pack fewer items into those bins with smaller capacity. See Figure 14B as an example, three items 3, 4 and 5 need to be packed into bins 1 and 2 when BinPacker processes node 5. If two items (items 3 and 5) are packed into bin 1 and only one (item 4) into bin 2, then we have m = 2 and n = 1 at node 4 ($m > n$). The packing strategy of BinPacker is reasonable because the capacity of bin 1 is larger than that of bin 2, which indicates that bin 1 should get more items than bin 2. If the strategy of BinPacker is that only one item is packed into bin 1 and another two into bin 2, then BinPacker will finally get the minimum path cover of the splicing graph. According to the sequencing depths of the junctions, items 3 and 5 should be packed into bin 1, and item 4 into bin 2. And when processing node 4, the item 4 packed into

bin 2 is replaced by two new items 6 and 7, with sizes 8 and 12. However, if we are forced to pack two items into bin 2 and another one into bin 1, we can see from the splicing graph that any packing strategies are not reasonable because the size of bin 2 is much smaller and only item 4 suits its size.

Theorem 2 The nodes previously processed will never be trapped again.

Proof The correctness of the theorem can be easily seen from the second constraint of quadratic programming (2) in the main article.□

Step 3. Bin packing by 0-1 quadratic programming. See the main article for details.

Step 4. Transformation into 0-1 ILP. It is proved by theorem 3 that the 0-1 quadratic programming in the main article can be equivalently transformed into a 0-1 linear programming by introducing a new variable x_{ijik} for each quadratic term $x_{ij} \cdot x_{ik}$ (or $x_{ik} \cdot x_{ij}$) in the objective function along with the constraints as follows:

$$\begin{cases} x_{ijik} \le x_{ij}, \quad \forall i = 1, \dots m, \quad 1 \le j \le k \le n \\ x_{ijik} \le x_{ik}, \quad \forall i = 1, \dots m, \quad 1 \le j \le k \le n \\ x_{ij} + x_{ik} - 1 \le x_{ijik}, \quad \forall i = 1, \dots m, \quad 1 \le j \le k \le n \\ x_{ijik} \in \{0,1\} \quad \forall i = 1, \dots m, \quad 1 \le j \le k \le n \end{cases}$$

Theorem 3 ***The transformed 0-1 integer linear programming is equal to the original 0-1 quadratic programming.***

Proof Denote by P the original 0-1 quadratic programming and by Q the transformed 0-1 integer linear programming. We prove that there is a one-to-one mapping f from the set of P's feasible solutions to that of Q's, and that in this mapping f, any feasible solution x of P has the same value of objective function as that of Q with the solution $x' = f(x)$.

For any solution x of P, $x' = f(x)$ is defined as follows. In x', we define x'_{ij} to be x_{ij} in x. For x'_{ijik}, we define $x'_{ijik} = 1$ if $x'_{ij} = x'_{ik} = 1$, and $x'_{ijik} = 0$ otherwise. Clearly, x' is a feasible solution of Q. If $x' = f(x)$, $y' = f(y)$ and $x' = y'$, which means that $x_{ij} = x'_{ij} = y'_{ij} = y_{ij}$, so it is clearly that $x = y$. On the other hand, under the added constraints, for any feasible solution x' of Q, we can define a variable x with $x_{ij} = x'_{ij}$, and x is clearly a feasible solution of P and $f(x) = x'$, which means that for any feasible solution x' of Q there is a feasible solution x of P such that $f(x) = x'$. This suggests that f is a one-to-one mapping from the feasible solution of P to that of Q.

Suppose that x is a feasible solution of P and $x' = f(x)$ is the corresponding solution of Q. The value of the objective function of P at x is z_P, and that of Q at x' is z_Q. From the definition of the mapping f we have that $x_{ij} = x'_{ij}$ and $x_{ij} \cdot x_{ik} = x'_{ijik}$ for any i, j, k. We have proved that $z_P = z_Q$, i.e., the 0-1 integer linear programming Q is equivalent to the original 0-1 quadratic programming P.□

BinPacker recovers an optimal set of full-length transcripts

Detailed steps of recovering an optimal set of full-length transcripts from the solutions of the

0-1 ILPs have been presented in the main article.

3. Supplementary Experiment

3.1 Tests on E.coli dataset

The E.coli dataset is adopted to evaluate the performance of the *de novo* assemblers on low complexity genome species without alternative splicing events. The E.coli data was collected from NCBI SRA database (Accession Code: SRR1931680). The criteria for evaluating the assemblers on the E.coli dataset are identical to what were used in the main article.

The sensitivities of BinPacker, Trinity and Bridger, which respectively recover 1452, 1437 and 1467 full-length transcripts out of 29975, 59576 and 46594 candidates, are higher than the other assemblers, except SOAPdenovo-Trans, which recovered 1560 full-length transcripts out of 160465 candidates (Figure A(i), shaded area) but at the cost of getting more false positives. For the accuracy, BinPacker performs much better than all the other assemblers in terms of both types of accuracy (Figures A(ii), A(iii), shaded area). IDBA-Tran performs very well in terms of reference true positive rate, even better than Trinity and Bridger, but it performs worse than Trinity and Bridger in terms of assembled true positive rate.

We also compute the sensitivity and accuracy distributions against recovered sequence length rates ranging from 80% to 100%. For the sensitivity distribution, the three curves of BinPacker, Bridger and Trinity are almost coincident with the highest sensitivity among all *de novo* assemblers, except SOAPdenovo-Trans, which outputs the most false positives (Figure A(i)). BinPacker keeps the highest accuracy for both types of accuracy in the whole interval [80%, 100%] (Figures A(ii), A(iii)). Therefore, we conclude from the comparison results that BinPacker is most stable as well on low complexity genome species without alternative splicing isoforms.

The computing resources, including running time and memory usage, were compared among the assemblers on the same server (Figure B). As expected, ABySS uses the least memory (Figure B(ii)), while SOAPdenovo-Trans takes the shortest time (Figure B(i)). As an exhaustive enumeration algorithm, Trinity consumes the largest memory and longest running time. BinPacker and Bridger require almost same memory, a little more than most of compared assemblers, but much less than Trinity (Figure B(ii)). As for the running time, BinPacker is faster than all assemblers but Oases and SOAPdenovo-Trans.

3.2 Tests on simulated dataset

The criteria for matching against reference transcripts on simulated dataset are identical to those explained in the main article.

3.2.1. Comparison of sensitivities and their distributions against recovered sequence length rates

Running all the eight *de novo* assemblers on the simulated dataset, we found that BinPacker reaches the highest sensitivity, recovering 7519 full-length transcripts from 21999 candidates, while Trinity recovers 7244 from 26924 and Bridger recovers 7014 from 21347. The rest of the assemblers all perform worse than any of the three (Figure 4A, shaded area). Trinity performs worse than BinPacker because Trinity uses an exhaustive enumeration algorithm to search for paths in *de Bruijn* graphs without using sequencing depth information in the searching process, which results in the increase of false positives and the decrease of true positives. Bridger performs worse than BinPacker is due to the facts: 1) the weights in the compatibility graph are defined a bit arbitrarily, and 2) a node with both in-edges and out-edges in the splicing graph will never be an end of a transcript. Stringtie performs best, outputting not only the largest number of full-length recovered reference transcripts but also the smallest number of false positives, showing that the reference genome is indeed helpful in transcriptome assembly.

To test the reliability of the *de novo* assemblers, we computed the sensitivity distributions against recovered sequence length rates ranging from 80% to 100%. As shown in Figure 4A, among the *de novo* assemblers, BinPacker keeps the highest sensitivity on the whole interval [80%, 100%]. Trinity and Bridger perform worse than BinPacker, but better than most of the other assemblers in the interval [90%, 100%] (Figure 4A). Therefore, we conclude from the comparison results among sensitivity distributions that BinPacker is most reliable among all the *de novo* assemblers we are comparing with.

3.2.2. Comparison of accuracies and their distributions against recovered sequence length rates

Comparison results demonstrate that BinPacker outperforms all the other *de novo* assemblers we are comparing with in terms of both types of accuracy (Figures 4B, 4C, shaded area). Among the other assemblers, Trinity reaches the highest reference true positive rate, while Bridger reaches the highest assembled true positive rate. Trans-ABySS performs better than ABySS for the reference true positive rate, but worse in terms of assembled true positive rate as it assembles more false positives. As expected, Stringtie has the highest accuracy of both types.

To test the reliability of these *de novo* assemblers, we computed the accuracy distributions against recovered sequence length rates ranging from 80% to 100%. As shown in Figures 4B and 4C, BinPacker keeps the highest accuracy in the whole interval [80%, 100%] among the *de novo* assemblers. For the other assemblers, Trinity reaches the highest reference true positive rate in the interval [90%, 100%], while Bridger shows the highest assembled true positive rate in the whole interval [80%, 100%].

Therefore we conclude that BinPacker is most reliable among all the *de novo* assemblers we are comparing with.

4. Installation and Usage Instructions of BinPacker

4.1 Installation from source code

1) Installing Boost

a) Download a version of boost and unpack it.

```
$ tar zxvf boost_1_47_0.tar.gz
```

b) Change to the boost directory and run ./bootstrap.sh.

```
$ cd boost_1_47_0
$ ./bootstrap.sh
```

c) Run

```
$ ./b2 install --prefix=<YOUR_BOOST_INSTALL_DIRECTORY>
```

Note: The default Boost installation directory is /usr/local. Take note of the boost installation directory, because you need to tell the BinPacker installer where to find boost later on.

2) Building BinPacker

a) Unpack the BinPacker and change to the BinPacker directory.

```
$ tar zxvf BinPacker_1.0.tar.gz
$ cd BinPacker_1.0
```

b) Configure BinPacker. If Boost is installed somewhere other than /usr/local, you will need to tell the installer where to find it using --with-boost option.

```
$ ./configure --with-boost=/path/to/boost/
```

c) Make BinPacker.

```
$ make
```

4.2 Quick installation

In order to make the BinPacker software more user-friendly and simpler to install, we have integrated the boost library and GLPK into our BinPacker software package. So users can install BinPacker through BinPacker_binary.tar.gz as follows.

a) Unpack the BinPacker and change to the BinPacker directory.

```
$ tar zxvf BinPacker_binary.tar.gz
$ cd BinPacker_binary
```

b) Make BinPacker.

```
$ ./update
```

4.3 Usage of BinPacker

** Required **

-s <string>: type of reads: (fa, or fq).

-p <string>: type of sequencing: (pair or single).

If paired_end reads:

-l <string>: left reads.

-r <string>: right reads.

If single_end reads:

-u <string>: single reads.

** Options **

-o <string>: name of directory for output, default: ./BinPacker_Out_Dir/

-m <string>: strand-specific RNA-Seq reads orientation, default: double_stranded_mode.

if paired_end: RF or FR;

if single_end: F or R.

-k <int>: length of kmer, default:25.

-g <int>: gap length of paired reads, default: 200.

-S <int>: minimum coverage of kmer as a seed, default: 2.

-E <float>: minimum entropy of kmer as a seed, default: 1.5.

-C <int>: minimum coverage of kmer used to extend, default: 1.

-N <float>: minimum entroy of kmer used to extend, default: 0.0.

-J <int>: minimum of the coverage of a junction, default: 2.

-v: report the current version of BinPacker and exit.

** Note **

A typical command of BinPacker might be:

BinPacker -s fq –p pair -l reads.left.fq -r reads.right.fq

(If your data are strand-strand, it is recommended to set -m option.)

4.4 Test the installation

Test data are provided with software distribution in the sample_test directory.

$ ./runMe.sh

5. Supplementary Figures

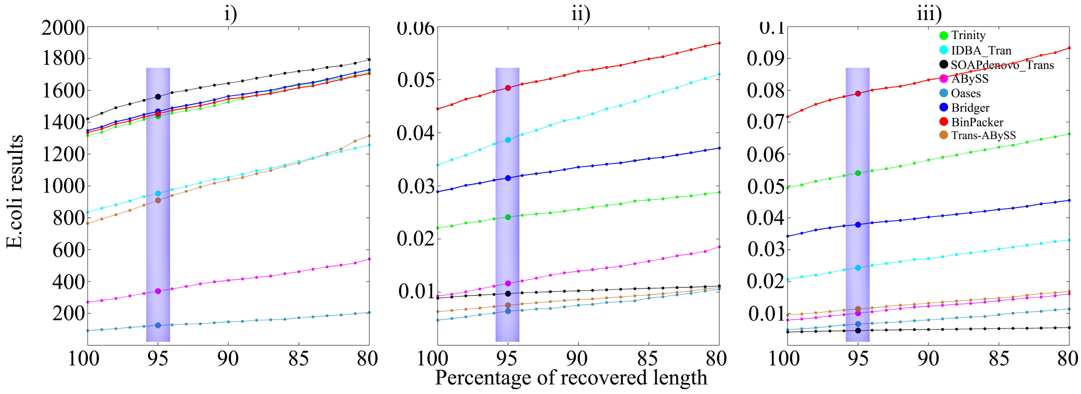

Figure A. Comparison among assemblers on E.coli dataset. (i) Recovered reference sensitivity and its distribution against recovered sequence length rates. The solid colored

circles in shaded areas represent the number of full-length recovered reference transcripts for different assemblers; (ii) Reference true positive rate and its distribution against recovered sequence length rates. The solid colored circles in shaded area represent the reference true positive rate for different assemblers; (iii) Assembled true positive rate and its distribution against recovered sequence length rates. The solid colored circles in shaded area represent the assembled true positive rate for different assemblers.

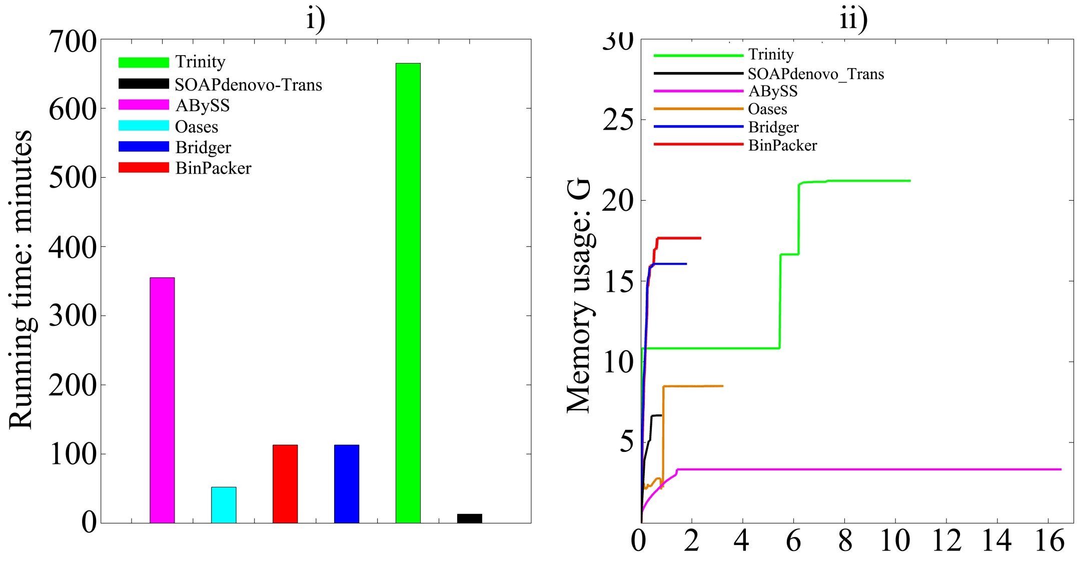

Figure B. Running time and RAM usage for each assembler on E.coli RNA-seq dataset. (i) running time of each assembler; (ii) memory usage of each assembler.

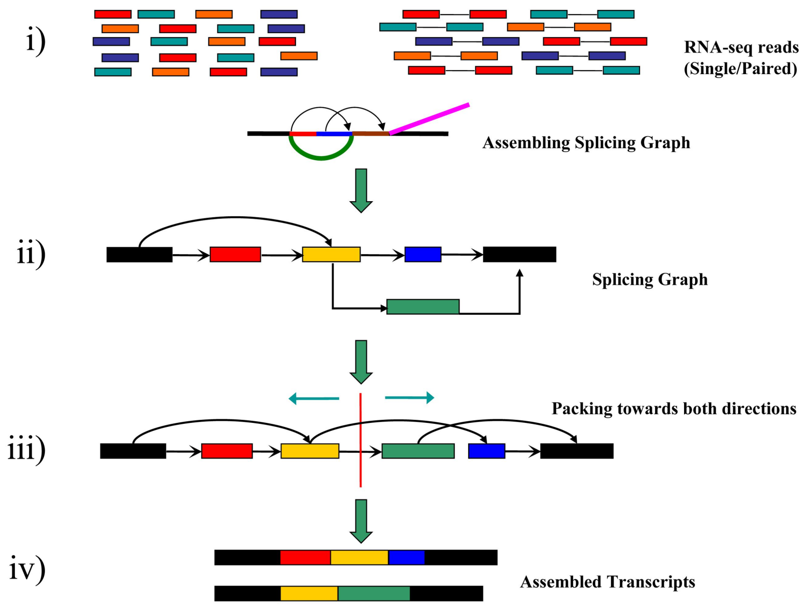

Figure C. Flowchart of BinPacker. (i) The algorithm takes RNA-seq reads (single or paired-end) to assemble splicing graphs, each of which provides a complete representation of all alternative splicing transcripts for each locus. (ii-iv) Each splicing graph is processed

independently. (ii) Each edge in a splicing graph represents one splice junction. (iii) A canonical graph, based on which the packing model is employed. (iv) A packing is applied to recover an optimal set of transcripts that could be tiled together through overlapping sequence reads and "explain" all observed junctions in a splicing graph.

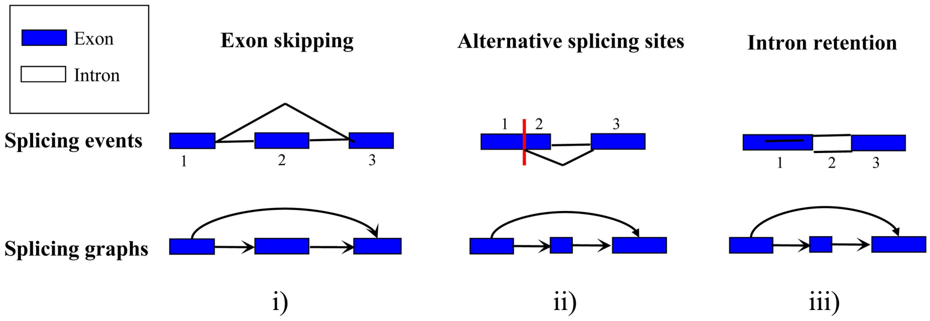

Figure D. Examples of splicing events and corresponding splicing graphs. (i) Exon skipping. Each node of a splicing graph is an actual exon. (ii) Alternative splicing sites. In the splicing graph of this case, two nodes (node 1 and node 2) correspond to one exon (the left one) of the gene (the vertical dash line shows the boundary). (iii) Intron retention. The possibly retended intron is represented as node in its splicing graph (e.g. node 2 in this example).

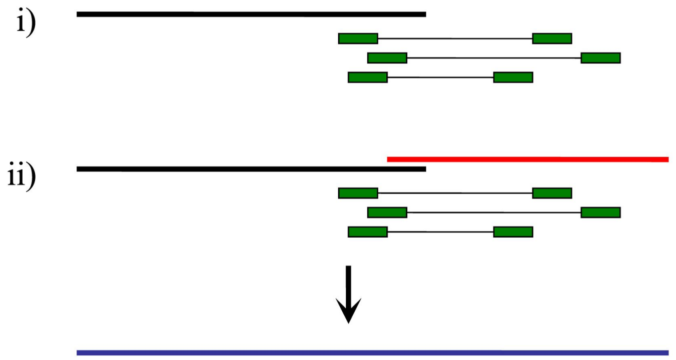

Figure E. Paired-end read information is used for constructing a complete trunk of the splicing graph. When the contig cannot be extended by overlapping *k*-mers, BinPacker (i) collects all paired-end reads with one end mapping the terminus of the contig and the other end mapping outside and (ii) generates a new contig starting from the end mapping outside of the current contig. Then these two contigs can be connected into a longer one.

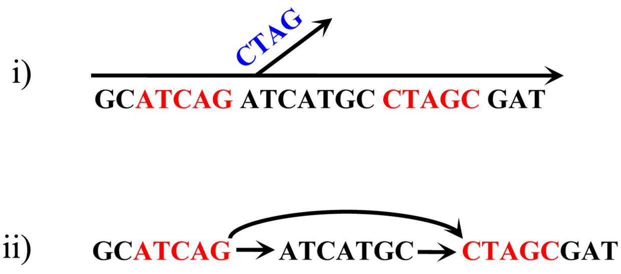

Figure F. Splicing graph construction. (i) Splicing graph after branch extension. The red k-mer (k=5) ATCAG on the left is a bifurcation 5-mer because there is an unused 5-mer TCAGC in the hash table that provides an alternative extension. Extend this 5-mer to a new contig until it cannot be further extended. We check the last 4-mer of this branch to see if there is a matching 4-mer in the current splicing graph. If so, another bifurcation 5-mer is found (e.g. the red 5-mer CTAGC). (ii) A modified splicing graph by merging the k-1 overlapping nucleotides (4-mer CTAG) and adding a new directed edge between two bifurcation k-mers.

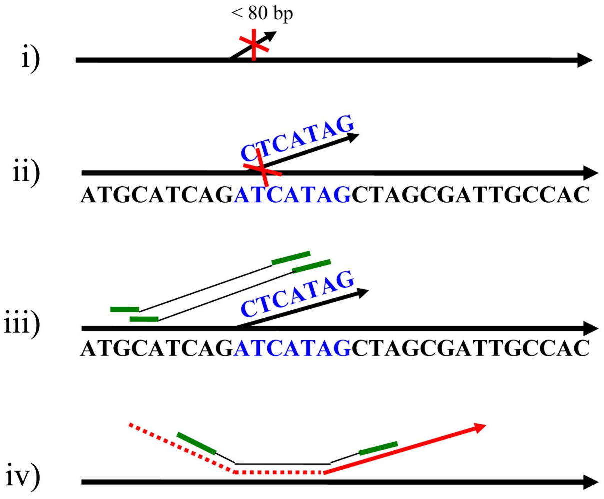

Figure G. Criteria used to decide if one potential branch is allowed to be added into the current splicing graph. (i) A branch must be long enough. If not, ignore it. (ii) A branch must be different from the corresponding part of the trunk. If not, ignore it. (iii) A branch that meets (i) and (ii) is allowed to be added into the graph if there exist at least two paired-end reads supporting it. (iv) Two paralogous genes, colored with red and green respectively, can be separated by paired-end read information.

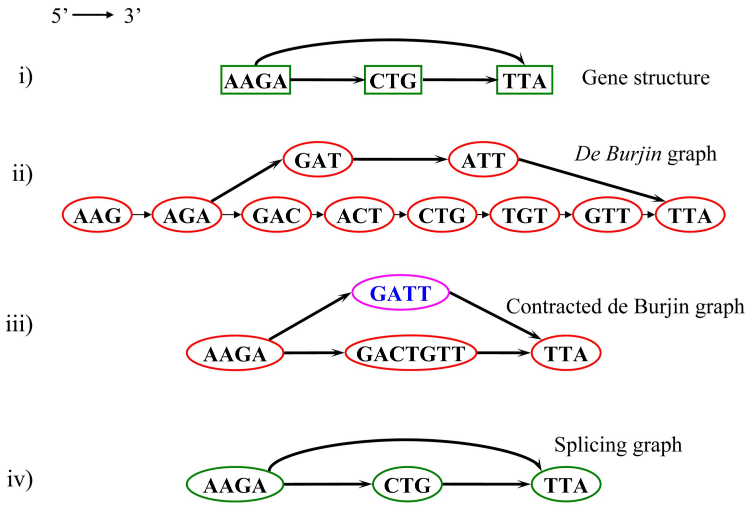

Figure H. One example shows that the splicing graph is different from contracted *de Bruijn* graph. (i) gene structure with two isoforms, (ii) *de Bruijn* graph, (iii) contracted *de Bruijn* graph, (iv) splicing graph.

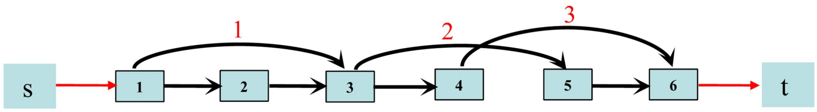

Figure I. An example shows compatible edges and incompatible edges in a canonical splicing graph, whose nodes are arranged linearly similar to a gene. In this graph, edge 1 and edge 2, edge 1 and edge 3, are compatible edges, while edge 2 and edge 3 are incompatible edges.

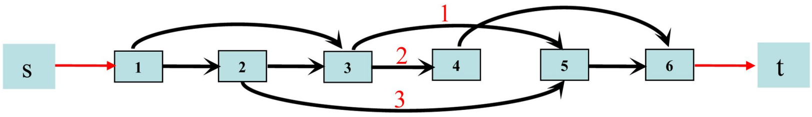

Figure J. An example shows the set of edges crossing two consecutive nodes. In this splicing graph, the edges crossing two consecutive nodes 3 and 4 are edge 1, edge 2 and edge 3.

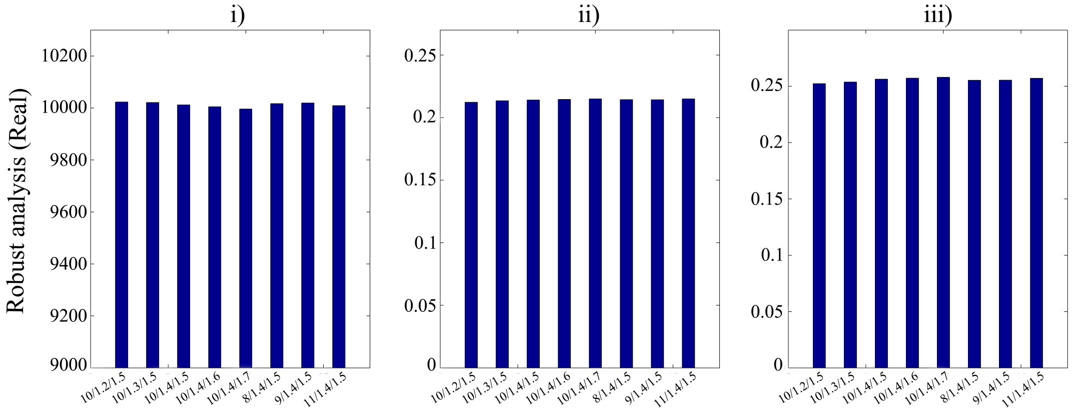

Figure K. Robustness analysis on real mouse dataset.

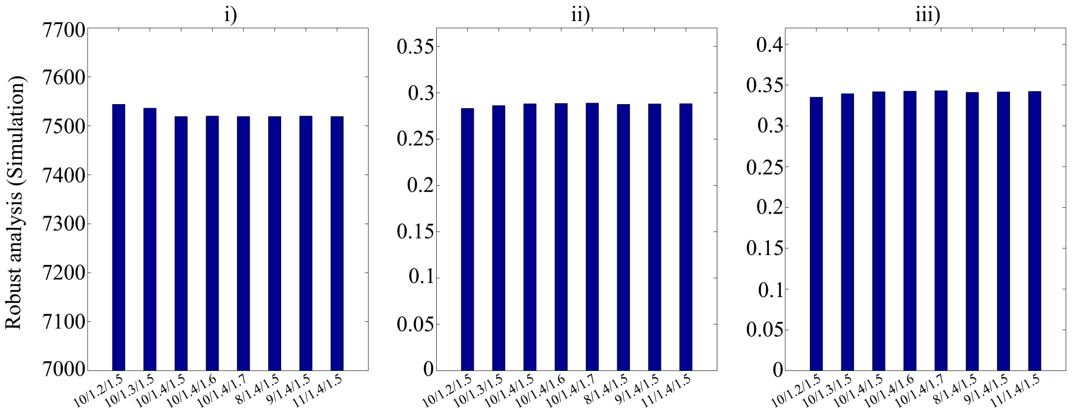

Figure L. Robustness analysis on simulated human dataset.

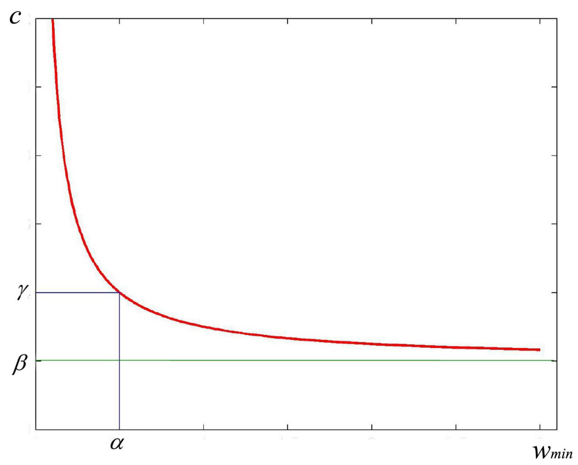

Figure M. Image of threshold c and its corresponding parameters α, β and γ.

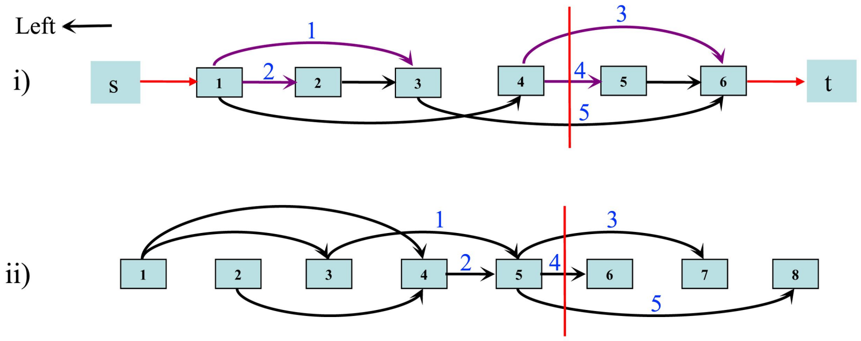

Figure N. Examples show the two cases leading to the occurrence of trap nodes. (i) The maximal set {edge 3, edge 4, edge5} is not maximum, while set {edge 1, edge 2, edge 3, edge 4} is. (ii) The numbers in parentheses represent the weights (sequencing depths) of the edges. We can see that items 3 and 5 should be packed into bin 1 and item 4 into bin 2. And when processing node 4, the item 4 packed into bin 2 is replaced by two new items 6 and 7, with sizes 8 and 12.

4. Supplementary Tables

Table A. Comparison of different RNA-seq assembly methods on dog data.

Method	Candidate transcripts	Full-length reconstructed reference transcripts	>= 80% length reconstructed reference transcripts
ABySS	29842	853	1386
Oases	47896	956	1545
Trinity	49311	1091	1657
BinPacker	33665	1149	1718
IDBA-Tran	32057	922	1524
SOAPdenovo-Trans	58842	930	1337
Cufflinks	60814	1498	4801

Table B. Comparison of different RNA-seq assembly methods on human data.

Method	Candidate transcripts	Full-length reconstructed reference transcripts	>= 80% length reconstructed reference transcripts
ABySS	36132	709	3791
Oases	60363	2424	9050
Trinity	54315	6122	14509
BinPacker	41691	5859	14328
IDBA-Tran	31095	3353	11606
SOAPdenovo-Trans	76611	1908	6740
Cufflinks	68067	7066	16915

Table C. Comparison of different RNA-seq assembly methods on mouse data.

Method	Candidate transcripts	Full-length reconstructed reference transcripts	>= 80% length reconstructed reference transcripts
ABySS	21993	4928	9701
Oases	42104	6941	13811
Trinity	78333	9599	15807
BinPacker	39060	10012	16270
IDBA-Tran	43717	3987	9098
SOAPdenovo-Trans	170830	2496	8552
Cufflinks	25108	8900	15341

Table D. Comparison of the number of recovered reference against recovered sequence length rates on dog data.

Method	BinPacker	ABySS	Trinity	Oases	IDBA-Tran	SOAPdenovo-Trans	Cufflinks
100%	636	427	610	517	480	536	685
99%	818	561	777	668	619	667	865
98%	919	659	871	754	708	751	990
97%	1005	734	949	830	789	824	1135
96%	1088	802	1031	898	866	883	1323
95%	1149	853	1091	956	922	930	1498
94%	1204	913	1148	1010	977	989	1672

93%	1260	954	1199	1060	1034	1029	1890
92%	1326	1011	1256	1121	1088	1067	2109
91%	1368	1044	1298	1164	1137	1104	2316
90%	1407	1088	1343	1201	1183	1130	2542
89%	1447	1124	1388	1247	1224	1159	2733
88%	1488	1155	1426	1286	1263	1181	2985
87%	1525	1197	1460	1327	1313	1213	3192
86%	1557	1220	1484	1357	1352	1237	3389
85%	1586	1248	1515	1391	1383	1254	3598
84%	1615	1284	1537	1423	1410	1270	3843
83%	1646	1312	1569	1462	1444	1289	4091
82%	1673	1344	1601	1489	1472	1308	4323
81%	1695	1369	1623	1513	1499	1325	4574
80%	1718	1386	1657	1545	1524	1337	4801

Table E. Comparison of the number of recovered references against recovered sequence length rates on human data.

Method	BinPacker	ABySS	Trinity	Oases	IDBA-Tran	SOAPdenovo-Trans	Cufflinks
100%	2616	287	2853	961	1189	721	3479
99%	3518	363	3718	1220	1572	944	4544
98%	4197	424	4432	1489	1975	1182	5316
97%	4742	503	4990	1770	2363	1411	5916
96%	5288	598	5522	2062	2808	1647	6486
95%	5859	709	6122	2424	3353	1908	7066
94%	6383	843	6638	2791	3864	2214	7617
93%	6942	1006	7171	3183	4385	2516	8214
92%	7485	1158	7725	3549	4891	2841	8810
91%	8068	1302	8294	3960	5476	3213	9449
90%	8644	1475	8856	4420	6046	3544	10108
89%	9173	1677	9348	4828	6582	3850	10782
88%	9734	1850	9924	5327	7150	4143	11451
87%	10294	2078	10493	5725	7726	4493	12124
86%	10836	2300	11029	6167	8230	4789	12717
85%	11411	2534	11581	6643	8781	5093	13430

84%	12016	2774	12169	7121	9330	5445	14139
83%	12550	2997	12738	7640	9894	5755	14794
82%	13141	3249	13333	8137	10472	6074	15498
81%	13728	3511	13910	8608	11050	6407	16213
80%	14328	3791	14509	9050	11606	6740	16915

Table F. Comparison of the number of recovered references against recovered sequence length rates on mouse data.

Method	BinPacker	ABySS	Trinity	Oases	IDBA-Tran	SOAPdenovo-Trans	Cufflinks
100%	5331	1408	5222	2563	1290	744	3984
99%	7362	2780	7174	3708	2251	959	6095
98%	8196	3530	7961	4608	2741	1191	7080
97%	8819	4050	8520	5455	3177	1576	7740
96%	9431	4506	9085	6231	3613	2021	8357
95%	10012	4928	9599	6941	3987	2496	8900
94%	10539	5341	10063	7578	4400	2994	9416
93%	11024	5714	10514	8214	4762	3479	9895
92%	11496	6088	10947	8771	5115	3955	10360
91%	11939	6436	11357	9251	5483	4388	10828
90%	12403	6790	11799	9790	5860	4839	11283
89%	12825	7149	12220	10281	6218	5277	11755
88%	13247	7458	12654	10715	6554	5673	12160
87%	13671	7779	13061	11162	6893	6083	12611
86%	14065	8077	13449	11543	7216	6452	13012
85%	14436	8344	13826	11924	7524	6832	13423
84%	14823	8624	14224	12316	7830	7176	13786
83%	15183	8907	14629	12709	8152	7518	14174
82%	15556	9186	15033	13087	8483	7867	14567
81%	15901	9428	15418	13455	8790	8211	14952
80%	16270	9701	15807	13811	9098	8552	15341

1. Grabherr MG, Haas BJ, Yassour M, Levin JZ, Thompson DA, et al. (2011) Full-length transcriptome assembly from RNA-Seq data without a reference genome. Nat

Biotechnol 29: 644-652.

2. CE S (1951) Prediction and entropy of printed English. Bell system technical journal: 50-64.
